# The H3K27me3-demethylase KDM6A is suppressed in breast cancer stem-like cells, and enables the resolution of bivalency during the mesenchymal-epithelial transition

**DOI:** 10.18632/oncotarget.19214

**Published:** 2017-07-10

**Authors:** Joseph H. Taube, Nathalie Sphyris, Kelsey S. Johnson, Keighley N. Reisenauer, Taylor A. Nesbit, Robiya Joseph, Geraldine V. Vijay, Tapasree R. Sarkar, Neeraja A. Bhangre, Joon Jin Song, Jeffrey T. Chang, Min Gyu Lee, Rama Soundararajan, Sendurai A. Mani

**Affiliations:** ^1^ Department of Translational Molecular Pathology, The University of Texas MD Anderson Cancer Center, Houston, Texas, USA; ^2^ Metastasis Research Center, The University of Texas MD Anderson Cancer Center, Houston, Texas, USA; ^3^ Department of Biology, Baylor University, Waco, Texas, USA; ^4^ Institute of Biomedical Sciences, Baylor University, Waco, Texas, USA; ^5^ Department of Integrative Bioscience, Texas A & M University, College Station, Texas, USA; ^6^ Depatment of Statistical Science, Baylor University, Waco, Texas, USA; ^7^ Center for Clinical and Translational Sciences, The University of Texas Health Science Center at Houston, Texas, USA; ^8^ Department of Integrative Biology and Pharmacology, The University of Texas Health Science Center at Houston, Texas, USA; ^9^ Department of Molecular and Cellular Oncology, The University of Texas MD Anderson Cancer Center, Houston, Texas, USA; ^10^ Center for Cancer Epigenetics, The University of Texas MD Anderson Cancer Center, Houston, Texas, USA

**Keywords:** epithelial-mesenchymal transition, mesenchymal-epithelial transition, KDM6A, bivalent genes, GSK-J4

## Abstract

The deposition of the activating H3K4me3 and repressive H3K27me3 histone modifications within the same promoter, forming a so-called bivalent domain, maintains gene expression in a repressed but transcription-ready state. We recently reported a significantly increased incidence of bivalency following an epithelial-mesenchymal transition (EMT), a process associated with the initiation of the metastatic cascade. The reverse process, known as the mesenchymal-epithelial transition (MET), is necessary for efficient colonization. Here, we identify numerous genes associated with differentiation, proliferation and intercellular adhesion that are repressed through the acquisition of bivalency during EMT, and re-expressed following MET. The majority of EMT-associated bivalent domains arise through H3K27me3 deposition at H3K4me3-marked promoters. Accordingly, we show that the expression of the H3K27me3-demethylase KDM6A is reduced in cells that have undergone EMT, stem-like subpopulations of mammary cell lines and stem cell-enriched triple-negative breast cancers. Importantly, KDM6A levels are restored following MET, concomitant with *CDH1*/E-cadherin reactivation through H3K27me3 removal. Moreover, inhibition of KDM6A, using the H3K27me3-demethylase inhibitor GSK-J4, prevents the re-expression of bivalent genes during MET. Our findings implicate KDM6A in the resolution of bivalency accompanying MET, and suggest KDM6A inhibition as a viable strategy to suppress metastasis formation in breast cancer.

## INTRODUCTION

The epithelial-mesenchymal transition (EMT) and the reverse process, the mesenchymal-epithelial transition (MET), are important cellular reprogramming events that endow tumor cells with the traits required to traverse the multiple steps of the metastatic cascade. The EMT program, which can be triggered by exposure to transforming growth factor beta (TGFB), converts polarized epithelial carcinoma cells into intrinsically motile and invasive spindle-shaped mesenchymal counterparts [1–3]. Additionally, EMT imparts cancer stem cell (CSC) characteristics, including intrinsic resistance to anoikis and genotoxic stresses as well as tumor-initiating capabilities [4, 5]. Consequently, EMT facilitates the initial steps of the metastatic cascade by promoting tumor cell detachment, invasion, and dissemination, whereas MET is evoked to restore the proliferative potential required for efficient colonization and formation of epithelial outgrowths at the distant site [6, 7].

The induction of EMT is accompanied by the downregulation of epithelial markers—most notably E-cadherin, the gatekeeper of the epithelial state (encoded by *CDH1*)—and the *de novo* expression of mesenchymal-associated genes [1]. The extensive changes in gene expression accompanying EMT/MET, coupled with the dynamic and reversible nature of the transitions between the epithelial and mesenchymal phenotypic states, suggest the involvement of epigenetic regulatory mechanisms in these processes [8–10]. Moreover, recent studies have begun to unravel the complexity of the epigenetic mechanisms that regulate stemness and the transition from a pluripotent to a differentiated state.

Post-translational modifications of histones are amongst the most extensively studied epigenetic mechanisms that can fundamentally alter gene expression. Indeed, the existence of a complex histone code has been proposed to explain how distinct combinations of histone modifications may converge to alter the transcriptional output of the underlying chromatin [11]. In particular, trimethylation of histone H3 at lysine 4 (H3K4me3) and lysine 27 (H3K27me3) has been associated with gene activation and silencing respectively [12–16]. The coexistence of these two conflicting activating and repressive marks within the same promoter, forming a so-called bivalent domain, was first described in human and mouse embryonic stem (ES) cells [17]. In ES cells, bivalent domains are prevalent in the promoters of differentiation-control genes and serve to maintain these genes in a silent but transcription-ready state, poised for lineage-specific upregulation or downregulation [17, 18]. Differentiation of ES cells into distinct lineages entails the resolution of bivalency by the removal of either the activating H3K4me3 mark, resulting in developmental silencing, or the repressive H3K27me3 mark, leading to gene activation [17, 18].

The bivalent chromatin configuration is also important in the context of CSC plasticity. In the plastic non-CSC subpopulations of human breast tumors, the promoter of ZEB1—a key EMT-inducing transcription factor—is bivalent, and resolves to an active H3K4me3-monovalent state, following exposure to TGFB, eliciting the induction of EMT and conversion to a CSC state [19]. Therefore, the resolution of bivalency is emerging as a critical epigenetic mechanism underpinning the switch between stem-like and differentiated cell states both during embryonic development and cancer progression.

We previously used genome-wide chromatin-immunoprecipitation followed by high-throughput sequencing (ChIP-Seq) to profile the patterns of H3K4me3 and H3K27me3 in immortalized human mammary epithelial cells (HMLE), and their counterparts induced to undergo EMT through ectopic expression of the EMT-inducing transcription factor Twist (HMLE-Twist) [20]. In addition to the extensive switching of monovalent H3K4me3 and H3K27me3 marks throughout the genome, we observed a significant enrichment of bivalent genes in mesenchymal HMLE-Twist cells relative to vector-transduced epithelial HMLE counterparts [20]. Here, we have focused on the subset of premarked monovalent H3K4me3-promoters, rendered bivalent and silenced through the addition of H3K27me3, that can be dynamically reactivated through subsequent H3K27me3 removal. Indeed, we found that modulation of H3K27me3 content is the predominant means of regulating gene expression during the transition from an epithelial to a mesenchymal state. The corollary of this observation is that the removal of the H3K27me3 mark from bivalent promoters may be a major route to the resolution of bivalency towards gene activation during EMT-reversal/MET.

To date, only two related H3K27me3-demethylases have been identified: lysine (K)-specific demethylase 6A (KDM6A)—also known as ubiquitously-transcribed X chromosome tetratricopeptide repeat protein (UTX1)—and KDM6B, also known as Jumonji-domain containing 3 (JMJD3) [21, 22]. Both KDM6A and KDM6B have been implicated in a wide range of differentiation processes as well as in cancer progression, but their respective transcriptional outputs are likely to be highly context-dependent [21, 23–25]. In fact, whereas KDM6B has been shown to promote EMT by removing the repressive H3K27me3 mark from the *SNAIL* (*SNAI1*) promoter [26], KDM6A has been implicated in the resolution of bivalency during retinoic acid-induced differentiation of mouse ES cells [27]. We therefore reasoned that KDM6A is a more likely candidate to demethylate H3K27me3 marks and drive the resolution of bivalency during MET.

Herein, we found that KDM6A is differentially expressed between stem and non-stem populations of breast immortalized and malignant cell lines, and that its protein levels decline during TGFB-induced EMT, with levels restored following TGFB-withdrawal and EMT-reversal/MET. In addition, we demonstrate that a significant proportion of EMT-associated bivalent genes encode effectors of cell differentiation, proliferation, phenotypic commitment and intercellular adhesions that are consequently silenced in the EMT/stem-like state. We further demonstrate that KDM6A plays a critical role in the MET-associated resolution and reactivation of these bivalent genes. These observations are consistent with a role for KDM6A in the resolution and activation of numerous bivalent genes, involved in proliferation and differentiation, to facilitate colonization during the latter stages of metastasis.

## RESULTS

### Genes rendered bivalent following EMT are predominantly associated with cell-cell adhesion, proliferation and developmental specification

We previously used genome-wide ChIP-Seq to profile the patterns of H3K4me3 and H3K27me3 in vector-transduced epithelial HMLE cells (hereafter HMLE-vector) versus mesenchymal HMLE-Twist cells, and detected a striking 2.7-fold increase in the number of bivalent genes in HMLE-Twist cells, compared to their epithelial counterparts [20]. Since genes may become bivalent by trimethylation of H3K4 at premarked H3K27me3 promoters, trimethylation of H3K27 at H3K4me3-monovalent promoters, or by the deposition of methyl groups at both sites, we classified the bivalent genes in HMLE-Twist cells into 4 groups, based on their pre-existing histone modifications in HMLE-vector cells: I) premarked with H3K4me3 in HMLE-vector cells that gain H3K27me3, II) premarked with H3K27me3 in HMLE-vector cells that gain H3K4me3, III) unmarked in HMLE-vector cells that acquire both H3K4me3 and H3K27me3, and IV) bivalent in HMLE-vector cells with no further change in status (Supplementary Table 1). Indeed, we found that the addition of H3K27me3 to premarked monovalent H3K4me3-promoters is the predominant modification contributing to the establishment of bivalency during the transition from an epithelial to a mesenchymal state, accounting for the *de novo* formation of 47% of bivalent domains (Supplementary Table 1) [20].

**Figure 1:**
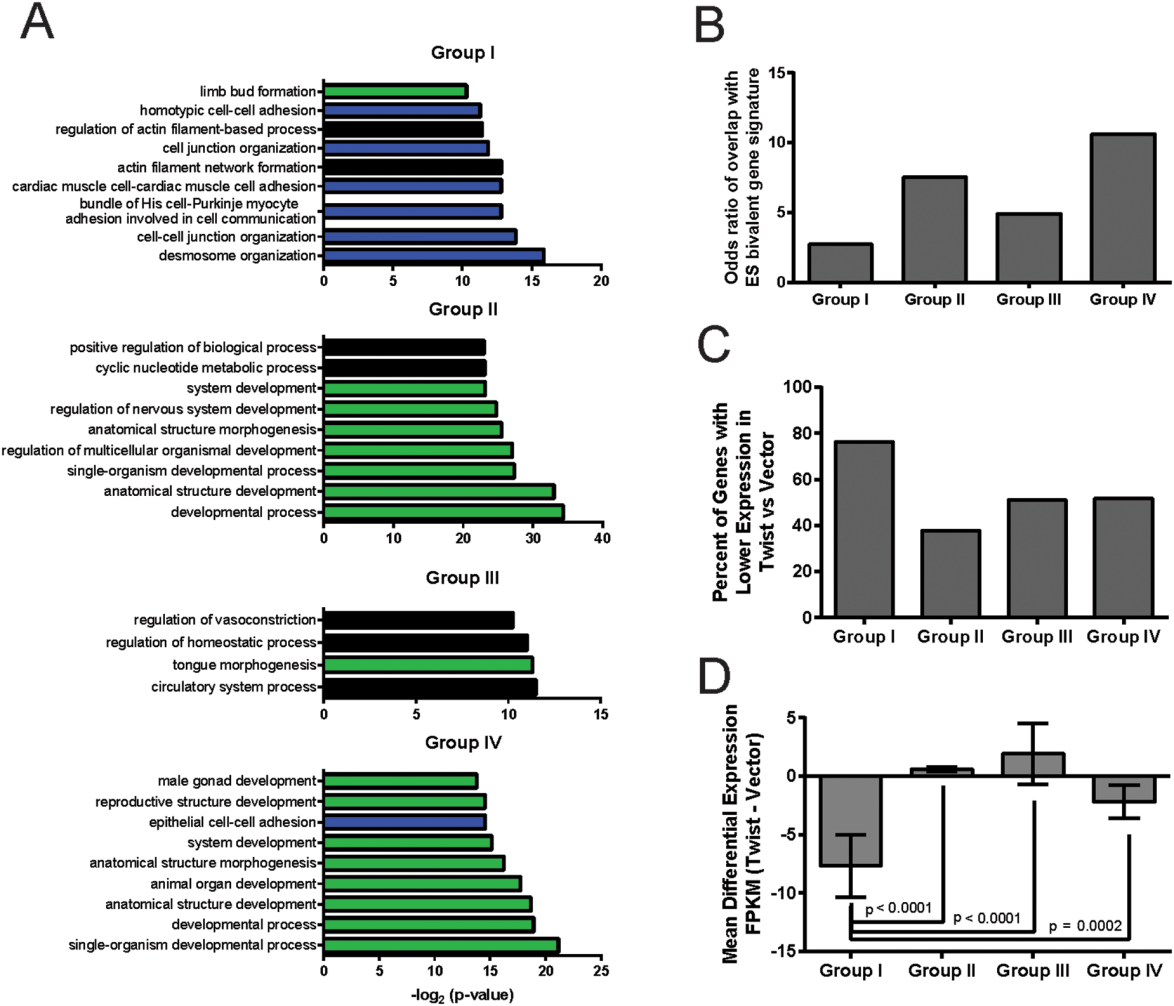
EMT-associated bivalent genes are enriched for regulators of proliferation, differentiation, and cell-cell adhesion, and are substantially repressed by addition of H3K27me3 Previously reported analyses of the epigenomes of HMLE-vector and HMLE-Twist cells, using ChIP-Seq for H3K27me3 and H3K4me3 and RNA-Seq, revealed that the number of bivalent genes (carrying both H3K4me3 and H3K27me3 marks within their promoters) is increased following Twist-induced EMT [20]. In the present study, all bivalent genes in HMLE-Twist cells were divided into 4 Groups: Group I: premarked with H3K4me3 in HMLE-vector cells that gain H3K27me3; Group II: premarked with H3K27me3 in HMLE-vector cells that gain H3K4me3; Group III: unmarked in HMLE-vector cells that acquire both H3K4me3 and H3K27me3; and Group IV: bivalent in HMLE-vector cells with no further change in status. **(A)** The top biological process gene ontology (GO) terms for each bivalent gene group were determined using GOrilla. The most enriched GO terms in each category are shown with their corresponding p-values, calculated as the exact minimum hypergeometric score using the GOrilla web server [70]. Green bars indicate categories relevant to developmental specification and blue bars indicate categories relevant to cell-cell adhesion and cytoskeletal architecture. Black bars indicate gene categories related to other biological processes. **(B)** The odds ratios of the overlap between the ES cell-derived and EMT-associated bivalent gene signatures were calculated, and are presented for each group of EMT-associated bivalent genes. **(C)** The percentage of bivalent genes in each group, exhibiting lower expression in HMLE-Twist cells compared to HMLE-vector cells, is graphed. Gene expression values are represented as Fragments Per Kilobase of transcript per Million mapped reads (FPKM), with data derived from RNA-Seq performed in [20]. **(D)** The cumulative gene expression differences (measured in FPKM) between HMLE-Twist and HMLE-vector cells were determined, and the means graphed. Values above 0 indicate that the average change in expression is higher in HMLE-Twist cells, compared to HMLE-vector cells. Error bars indicate the standard error of the mean. The p-values were determined by comparison of ranks by the Mann-Whitney test for non-parametric data.

In order to understand which biological processes may be regulated through the establishment of bivalency following EMT, we determined the enrichment for specific gene ontology terms in each category through gene ontology analysis. Strikingly, all 4 categories of bivalent genes are enriched for genes regulating development, cell fate specification and differentiation (Figure 1A, green bars). Examples of genes in these categories include transcription factors and signaling molecules such as *SOX4* and *SOX9* in Group I, *WNT1* and *WNT2* in Group II, *BMP4* and *NGFR* in Group III, and *PITX1* and *FGF9* in Group IV. Notably, the subset of genes that acquires bivalent status through the addition of H3K27me3 following EMT (Group I) is particularly enriched for genes involved in cell-cell adhesion and cytoskeletal architecture (Figure 1A, blue bars), consistent with the reduction of intercellular adhesions and the acquisition of intrinsic motility following EMT. Examples of Group I genes involved in the regulation of actin cytoskeletal integrity and cell-cell adhesion include genes encoding the actin-binding protein *COBLL1*, the desmosome components *DSG2* and *DSP*, and the constituent of adherens junctions *CADM1*. The finding that the combination of histone modifications, deposited at promoters rendering them bivalent, can classify the underlying genes into functionally distinct categories suggests that histone modifiers are selectively deployed to silence coordinated gene networks during EMT.

Since, in pluripotent ES cells, bivalent domains mark the promoters of genes involved in development and differentiation for silencing, we next examined the overlap between the sets of bivalent genes generated via EMT and genes identified as bivalent in ES cells. All 4 groups of bivalent genes, identified in our study, overlap at a statistically significant level (p<0.001 by Fisher’s exact test) with the ES cell-derived bivalent gene signature [17]. In particular, the two groups of bivalent genes most associated with developmental specification (Group II, bivalent through acquisition of H3K4me3 and Group IV, pre-existing bivalent marks) show the highest odds ratios of overlap with the ES cell-derived bivalent gene signature [17] (Figure 1B), underscoring the similarities in the epigenetic regulation of the EMT and ES cell stemness modules. It is interesting to note that the Group IV bivalent promoters, unaffected by EMT, correspond most closely with those that are also bivalent in ES cells, supporting the notion that a subset of bivalent promoters are maintained in a ‘poised’ state despite a multitude of both differentiation and de-differentiation signals. Our results suggest that these constitutively bivalent Group IV genes encode products pertaining to a high degree of differentiation and specialized functions (e.g. *LHX4*, *LHX9*, *LRRC6*, *PFKP*, *PPFIBP2*, *MEGF11*, *SEMA5B*), or genetic programs that are suppressed under normal physiological conditions such as the response to genotoxic stresses and cell death pathways (e.g. *TXNRD3*, *CERKL*, *SFXN1*, *PYCARD*, *PDRG1*, *NOC2L*). Conversely, the genes in Group I show the least significant overlap with the ES cell-derived bivalent gene signature and encompass components of cell adhesion complexes and the cytoskeleton that play a prominent role in organizing cellular architecture and intrinsic motility. Therefore, we reasoned that these genes likely represent an EMT/MET-relevant structural gene module that regulates cell shape, cell-cell adhesion, and migration.

We next examined the differences in gene expression between each of the four groups of genes which became bivalent through distinct histone modification events during EMT. Consistent with our expectations, 69.4% (256/369) of the bivalent genes that gained the H3K27me3 mark were expressed at a lower level in their bivalent state in HMLE-Twist cells, compared to their monovalent H3K4me3 state in HMLE-vector cells (Figure 1C). In fact, further analysis demonstrates that the cumulative gene expression changes among genes in Group I are significantly more dramatic than the other groups (II-IV) and, overall, tend towards lower expression in cells induced to undergo EMT (HMLE-Twist) compared to epithelial counterparts (HMLE-vector), as might be expected due to the addition of the H3K27me3 repressive mark to the active H3K4me3 monovalent configuration, rendering the gene repressed/bivalent (Figure 1D). Given the significant differential expression of Group I genes between the epithelial and mesenchymal states, the relatively small overlap with the ES cell bivalent signature, and the enrichment of biological pathways involved in the regulation of cell adhesion and cell-cell communication in the Group I gene dataset, we next focused on the modulation of H3K27me3 as a major factor regulating the formation of bivalent domains and the silencing of gene expression following EMT.

### The H3K27me3-demethylase KDM6A is dynamically regulated during EMT/MET and is differentially expressed in mesenchymal stem-like and non-stem cells

It is known that the resolution and activation of bivalent genes can promote differentiation in ES cells [28–30]. Given that MET at least partially restores differentiated epithelial characteristics to cells that have previously undergone EMT, and that our findings implicate the acquisition of H3K27me3 as a key modification leading to EMT-associated bivalency, we reasoned that an H3K27me3-demethylase activity might be suppressed during EMT, and reinstated during MET, thus enabling the resolution of bivalency. Therefore, we hypothesized that the activity and/or expression of the H3K27me3-demethylases KDM6A and KDM6B might be decreased following EMT, allowing the *de novo* accumulation of H3K27me3 and/or the retention of premarked H3K27me3 at bivalent domains.

**Figure 2:**
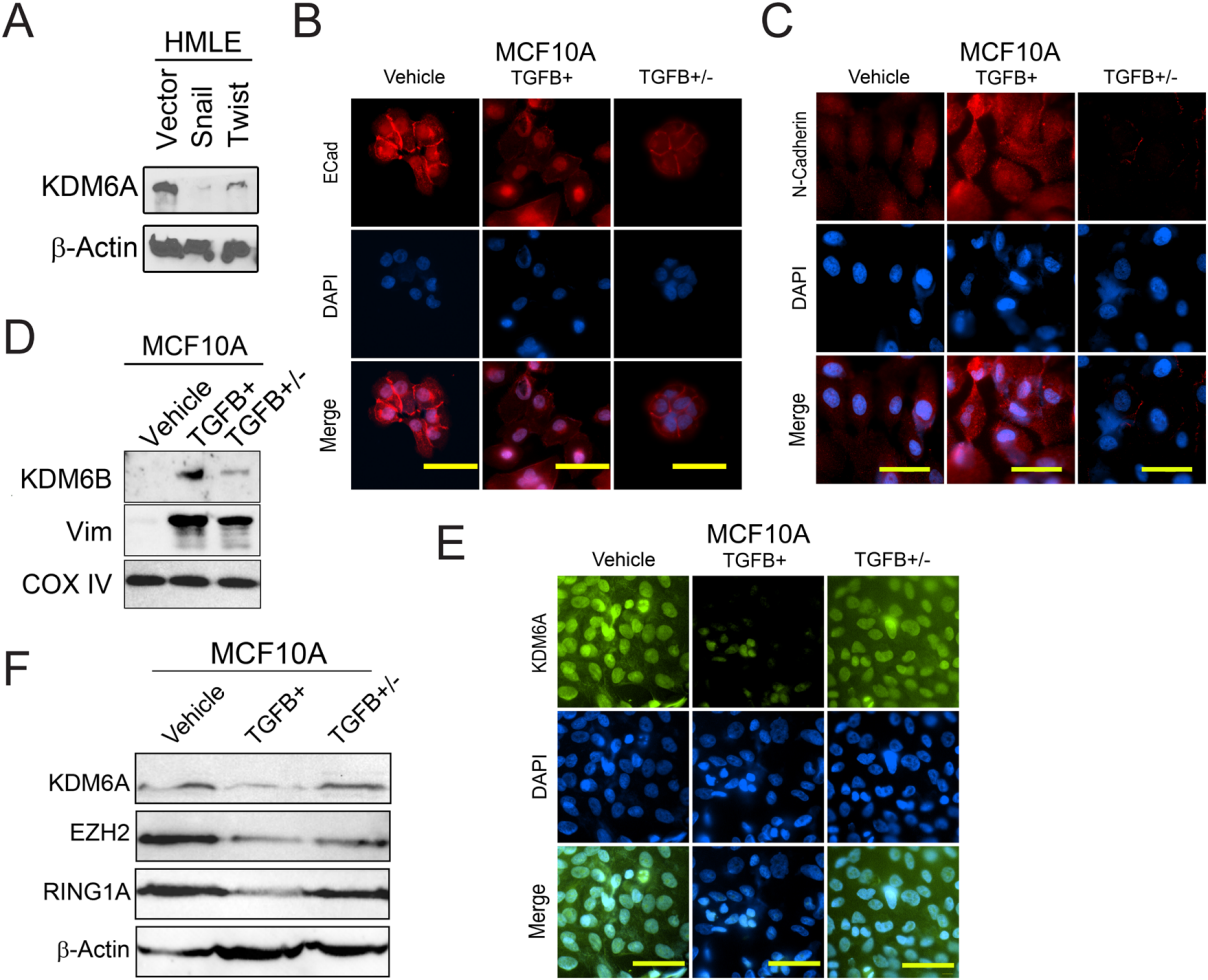
KDM6A protein levels are decreased following EMT and upregulated during MET **(A)** HMLE cells were transduced with either an empty vector (HMLE-Vector), or genes encoding the EMT-inducing transcription factors Snail (HMLE-Snail) or Twist (HMLE-Twist) [4]. Whole-cell lysates were analyzed by immunoblotting for KDM6A. β-actin was used as a loading control. **(B-F)** MCF10A cells were treated with sterile 4 mM HCl containing 0.1% bovine serum albumin (Vehicle) or TGFB for 5 days (TGFB+). In parallel, MCF10A cells were exposed to TGFB for 5 days in order to induce EMT, and subsequently subjected to TGFB-withdrawal, for a further 5 days, to elicit MET (TGFB+/-). (B-C) MCF10A cells, treated as described, were fixed and immunostained for E-cadherin (B) and N-cadherin (C). Nuclei were counterstained with DAPI (blue). Scale bars, 50 μm. (D) MCF10A cells, treated as described, were analyzed by immunoblotting for KDM6B and vimentin (Vim). COX IV was used as a loading control. (E) MCF10A cells, treated as described, were fixed and immunostained for KDM6A. Nuclei were counterstained with DAPI (blue). Scale bars, 50 μm. (F) Cells were analyzed by immunoblotting for KDM6A, EZH2 and RING1A. β-actin was used as a loading control.

We first investigated the expression of KDM6A in epithelial HMLE-vector cells in comparison with their mesenchymal counterparts overexpressing the EMT-inducing transcription factors Twist (HMLE-Twist) and Snail (HMLE-Snail). Using immunoblotting, we detected markedly reduced KDM6A protein levels in mesenchymal HMLE-Snail and HMLE-Twist cells compared to the epithelial HMLE-vector cells (Figure 2A). In addition, we observed intense immunostaining for KDM6A in the cytoplasm and nuclei of epithelial HMLE-vector cells, compared to the decreased KDM6A staining intensity of HMLE-Twist cells, most notably reduced in the nuclear compartment (Supplementary Figure 1A). We verified that HMLE-vector cells stained positive for E-cadherin, as expected, whereas HMLE-Twist cells were devoid of this epithelial marker, consistent with their mesenchymal phenotype (Supplementary Figure 1B). Interestingly, Twist1 has been shown to bind to a sequence 31 kb upstream of the *Kdm6a* gene in the context of mouse limb bud and endocardial cushion tissues [31]. However, the functional relevance of Twist occupancy, at this site, to *KDM6A* gene expression has yet to be ascertained. Nevertheless, our data are consistent with the notion that Twist may repress *KDM6A* transcription.

We also investigated the expression of KDM6A following TGFB-treatment of the E-cadherin^+ve^ mouse mammary tumor cell line, 4T1 [32]. As visualized by phase-contrast microscopy, treatment of 4T1 cells with TGFB over 5 days, elicited a marked reduction in cell-cell contacts and the acquisition of an elongated spindle-shaped morphology, consistent with the induction of EMT (Supplementary Figure 2A). Whereas KDM6A was readily detected in vehicle-treated 4T1 cells, we observed a dramatic reduction in KDM6A immunostaining following TGFB treatment, concurrent with the loss of the epithelial marker E-cadherin (Supplementary Figures 2B and 2C). These findings are consistent with an association of KDM6A expression with the epithelial phenotype, and its exclusion from the mesenchymal lineage. Moreover, these expression patterns suggest that KDM6A may function selectively/primarily in the epithelial setting, with the corollary that KDM6A depletion during EMT may account for the marked increase in bivalent domains detected in the genome of HMLE-Twist cells.

Since our findings indicate that KDM6A protein levels are depleted following EMT, we next sought to test whether KDM6A levels are reinstated during MET to facilitate the resolution of bivalency and the activation of genes involved in differentiation. For this, we utilized a physiologically-relevant model of TGFB-induced EMT in spontaneously immortalized mammary epithelial MCF10A cells, followed by EMT-reversal/MET elicited by TGFB-withdrawal. In this model, we observed diminished levels of membrane-localized E-cadherin in TGFB-treated MCF10A cells, compared to vehicle-treated epithelial counterparts, which exhibited a predominantly membranous expression pattern (Figure 2B). Moreover, TGFB treatment of MCF10A cells elicited an increase in the expression of the mesenchymal markers N-cadherin (Figure 2C) and vimentin (Figure 2D and Supplementary Figure 3). Conversely, TGFB-withdrawal was accompanied by restoration of E-cadherin to the cell membrane, and a decrease in the expression of vimentin and N-cadherin, consistent with EMT reversal/MET and re-differentiation towards an epithelial phenotype (Figures 2B-2D and Supplementary Figure 3). As expected, KDM6A protein levels decreased upon TGFB treatment (Figures 2E and 2F). Interestingly, upon TGFB-withdrawal/MET, KDM6A protein levels were restored to levels commensurate with those of vehicle-treated cells (Figures 2E and 2F). In contrast to our results for KDM6A, we observed increased expression of the related H3K27me3-demethylase KDM6B in TGFB-treated MCF-10A cells that have undergone EMT, relative to vehicle-treated epithelial counterparts (Figure 2D). This finding is consistent with a recent study showing that KDM6B expression is induced during EMT and, in fact, that KDM6B promotes EMT progression by de-repressing the *SNAI1* promoter, through demethylation of the H3K27me3 mark, thus driving *Snail* expression [26]. Overall, our data indicate that KDM6B and KDM6A protein levels are inversely regulated during EMT, commensurate with decreased E-cadherin protein levels and increased mesenchymal marker expression. Conversely, during TGFB-withdrawal/MET, KDM6A protein levels are restored to the levels of vehicle-treated cells, concurrent with a decrease in KDM6B levels.

One potential explanation for the accumulation of H3K27me3 at bivalent genes could be an increase in the expression of components of the Polycomb Repressor Complex 2 (PRC2), which mediates methylation at H3K27 [33]. To determine if this is the case, we used our dynamic EMT/MET model to profile the expression of EZH2, the histone methyltransferase that functions as the catalytic subunit of PRC2, and RING1A which acts as a transcriptional repressor within the PRC2 complex. Surprisingly, we found that the protein levels of both EZH2 and RING1A were decreased during TGFB-induced EMT in MCF10A cells (Figure 2F). This unexpected finding underscores the significance of the reduction in KDM6A expression for the establishment of bivalency during EMT. Thus, the accumulation of H3K27me3 at bivalent genes, during EMT, is not accomplished through the increased activity of a molecular writer driving increased deposition, but rather through the absence of KDM6A demethylase activity, evidenced by the persistence of pre-existing or newly-deposited marks at H3K4me3-premarked promoters.

**Figure 3:**
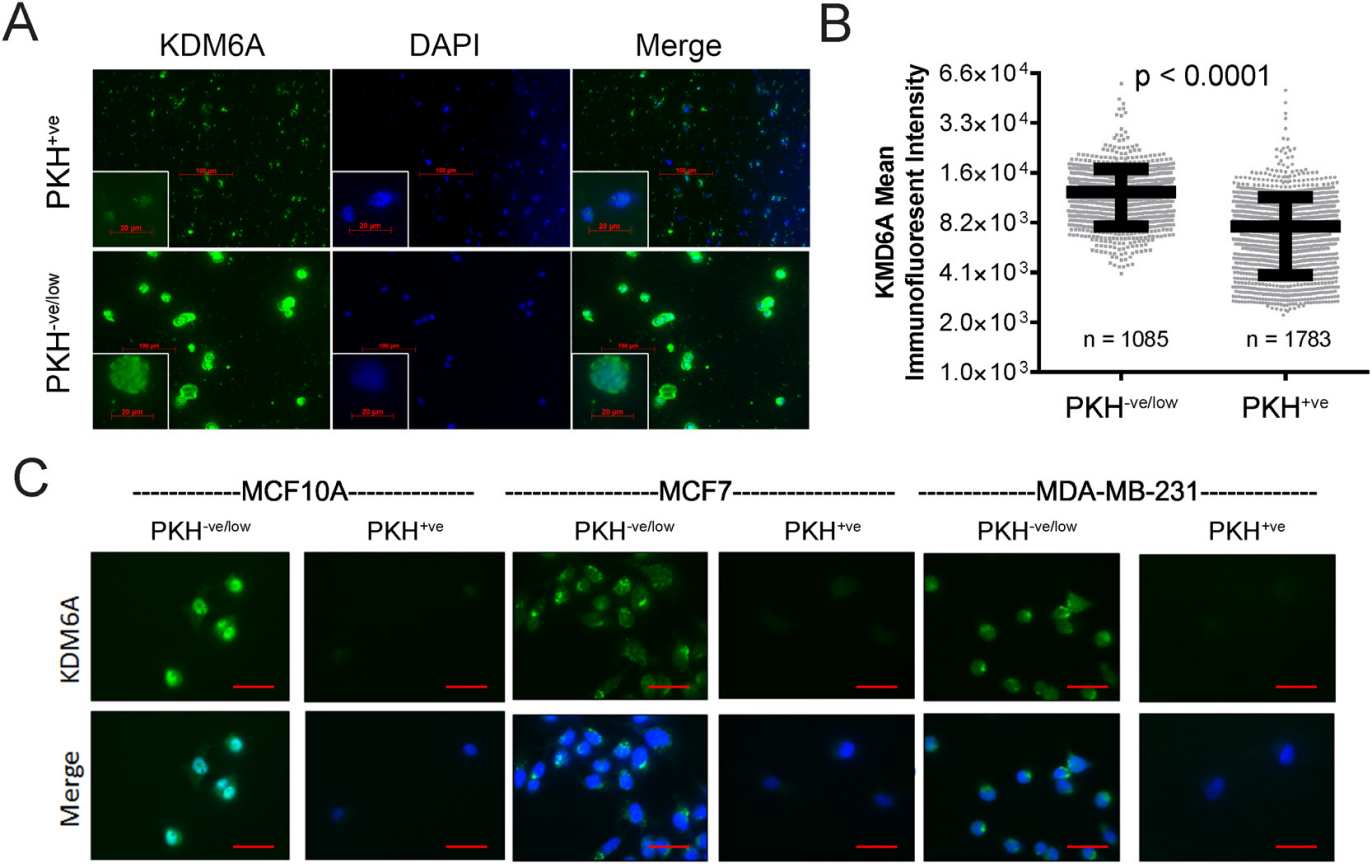
Stem cell-enriched subpopulations of mammary cell lines express lower KDM6A levels compared to non-stem counterparts **(A)** MCF10A cells were labeled with PKH26 and sorted by FACS into PKH26^-ve/low^ and PKH26^+ve^ populations, and subsequently processed for KDM6A immunostaining. Images from random fields were captured using a Cytation 3 automated digital microscope. Representative images of KDM6A immunostaining (green) in PKH26^-ve/low^ and PKH26^+ve^ MCF10A cells, centrifuged onto slides by cytospin, are shown. Nuclei were counterstained with DAPI (blue). Scale bars, 100 μm. The insets represent magnified images of selected areas. Scale bars, 20 μm. **(B)** Image analysis was performed to determine the relative intensity of the immunofluorescence signal within the nuclei of individual cells. The mean immunofluorescent intensity of KDM6A staining was plotted for the respective PKH26^-ve/low^ and PKH26^+ve^ subpopulations. n equals the number of nuclei analyzed for each MCF10A subfraction. P-values were calculated using an unpaired, two-tailed t-test. **(C)** Cells from the indicated cell lines were sorted into PKH26^-ve/low^ and PKH26^+ve^ subfractions by FACS. Immunofluorescent staining for KDM6A was performed on PKH26^-ve/low^ and PKH26^+ve^ cells that were seeded onto glass coverslips after sorting. Nuclei were counterstained with DAPI (blue). Scale bars, 50 μm.

We and others have shown that the stem cell-enriched fractions of established mammary cell lines exhibit mesenchymal characteristics [4, 5], suggesting that these stem-like cell subsets may also express lower levels of KDM6A, compared to more differentiated subpopulations. To test this, we used the lipophilic fluorescent membrane-intercalating dye, PKH26, as a functional means to enrich for mammary stem cells [34]. PKH26 labels all cells evenly in the initial population, but is progressively diluted out from highly proliferative cells during cell division. Thus, PKH26 is selectively retained in the membranes of the relatively slow-cycling cells, a population that includes mammary stem cells, which can therefore be recovered using fluorescence-activated cell sorting (FACS) [34–36]. To quantify the differences in KDM6A expression between the stem-like and differentiated cell subsets, we sorted PKH26^+ve^ and PKH26^-ve/low^ MCF10A cells, 7 days following PKH26 addition. Given the small numbers of PKH26^+ve^ stem-like cells compared to the bulk PKH26^-ve/low^ populations, we centrifuged FACS-sorted PKH26^-ve/low^ and PKH26^+ve^ cell suspensions onto glass slides by cytospin and performed immunofluorescent staining for KDM6A (Figure 3A). To quantify our results, the intensity of KDM6A staining within a nucleus stained with 4', 6-diamidino-2-phenylindole (DAPI) was measured using Gen5 image analysis software. The mean intensity of KDM6A staining was significantly lower in the stem-like PKH26^+ve^ MCF10A cell fraction compared to the PKH26^-ve/low^ MCF10A subpopulation (Figure 3B). To expand our findings to additional cell lines, MCF7 and MDA-MB-231 cells were sorted for PKH26^-ve/low^ and PKH26^+ve^ cells, with MCF10A cells serving as a control. In order to better visualize the KDM6A staining pattern, we performed immunofluorescence using sorted cells deposited directly onto glass coverslips, without centrifugation. In line with our previous observations, KDM6A was noticeably absent from the nuclei of the stem-cell enriched PKH26^+ve^ subpopulations from MCF10A cells as well as MCF7 and MDA-MB-231 breast malignant cell lines (Figure 3C). In contrast, we observed prominent nuclear KDM6A staining in the more differentiated PKH26^-ve/low^ subpopulations (Figure 3C). Collectively, these results show that the slow-cycling, stem cell-enriched subpopulations of multiple mammary cell lines display significantly diminished KDM6A staining compared to the faster-cycling, differentiated subpopulations.

### KDM6A occupancy inversely correlates with H3K27me3 levels at the *CDH1* locus

**Figure 4:**
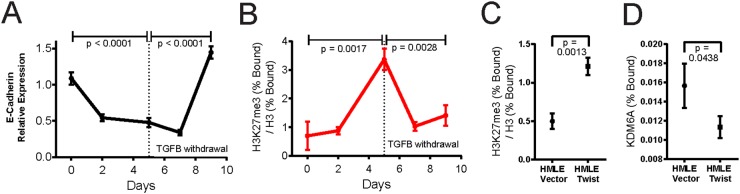
H3K27me3 deposition is reversible upon MET and revokes CDH1/E-cadherin silencing MCF10A cells were treated with TGFB (5 ng/ml) for five days to induce EMT, followed by five days of culture without TGFB, eliciting MET. As a control, MCF10A cells were treated with vehicle during the first 5 days of the timecourse. **(A)** qRT-PCR was used to monitor *CDH1*/E-cadherin mRNA levels at the indicated timepoints. *GAPDH* was used as the reference gene to normalize the variability in template loading. **(B)** Chromatin immunoprecipitation-PCR (ChIP-PCR) analysis was done on the transcription start site of the *CDH1* gene to determine the relative enrichment of H3K27me3 at this locus in MCF10A cells, harvested at the indicated timepoints. **(C, D)** ChIP-PCR analysis was used to determine the relative enrichment of H3K27me3 (C) and the KDM6A occupancy (D) at the *CDH1* transcription start site, following immunoprecipitation of lysates from HMLE-vector and HMLE-Twist cells with the respective antibodies. For H3K27me3, values were normalized to immunoprecipitation of total H3. The background enrichment for a subtype-control IgG antibody was subtracted. P-values were calculated using an unpaired, two-tailed t-test.

Given that the EMT and MET programs unfold over time, and are underpinned by gradual changes in gene expression with *CDH1*/E-cadherin repression widely regarded as the hallmark of EMT, we next undertook to conduct a timecourse analysis of the changes in H3K27me3 occupancy at the *CDH1*/E-cadherin promoter. For this, we transiently treated MCF10A cells with TGFB over 5 days, followed by TGFB-withdrawal for a further 5 days, and harvested samples for quantitative RT-PCR analysis and ChIP assays at appropriate timepoints. Consistent with the induction of EMT, TGFB treatment of MCF10A cells elicited a gradual decrease in the expression of the *CDH1* gene. Conversely, TGFB-withdrawal was accompanied by a progressive increase in *CDH1* transcript levels, signifying a return to an epithelial phenotype (Figure 4A). Accordingly, we found that H3K27me3 was enriched at the transcription start site of the *CDH1*/E-cadherin promoter following TGFB treatment, consistent with the notion that accumulation of H3K27me3 drives *CDH1* repression during EMT. Following TGFB-withdrawal, the levels of H3K27me3 at the *CDH1* transcription start site were diminished, thus establishing a transcription-permissive state (Figure 4B).

Additionally, we sought to determine whether KDM6A directly regulates *CDH1* expression and if selective enrichment of H3K27me3 at the *CDH1* promoter is associated with *CDH1*/E-cadherin repression in the mesenchymal state. For this, we performed ChIP assays, using HMLE-vector and HMLE-Twist cells, which exhibit epithelial and mesenchymal characteristics respectively. As predicted, H3K27me3 was detected at 2.4-fold higher levels at the *CDH1* promoter in HMLE-Twist cells, which do not express E-cadherin (Figure 4C), compared to HMLE-vector cells. On the other hand, KDM6A binding to the *CDH1* promoter was detected to a much higher degree in HMLE-vector cells, suggesting a role for KDM6A in demethylating H3K27me3 to enhance/maintain basal E-cadherin expression levels (Figure 4D). Taken together, these data support a dynamic role for KDM6A in the removal of the H3K27me3 repressive mark to maintain the *CDH1* promoter in a transcriptionally-active state, with the corollary that the loss of KDM6A activity, during EMT, will lead to accumulation of H3K27me3 and silencing of *CDH1*.

### Inhibition of KDM6A activity during MET blocks reactivation of bivalent genes

**Figure 5:**
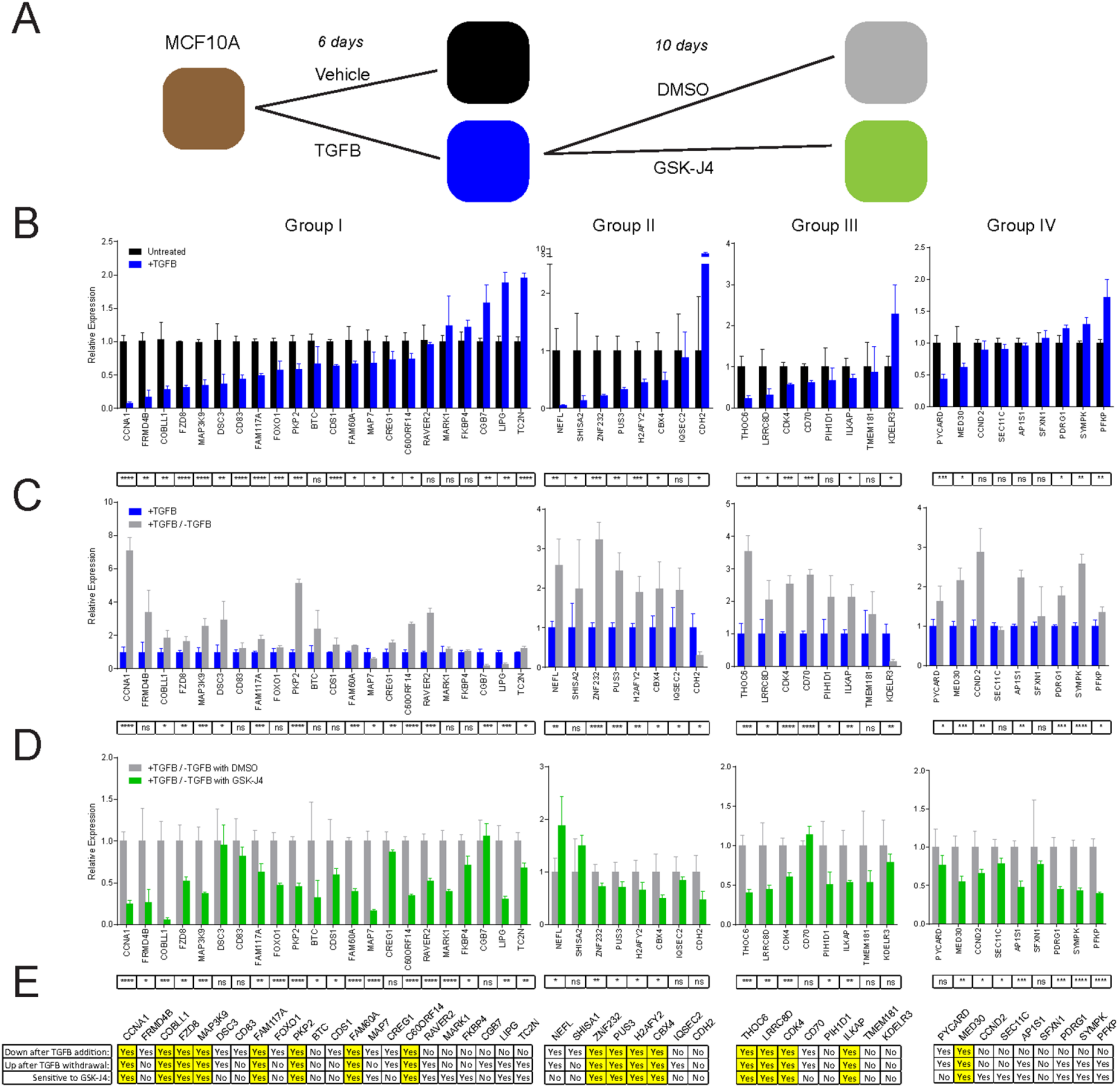
KDM6A inhibition by GSK-J4 blocks reactivation of bivalent genes **(A)** RT-PCR for the indicated genes was performed on RNA extracted from MCF10A cells treated with vehicle or TGFB (2.5 ng/ml), and TGFB-treated MCF10A cells, subsequently subjected to TGFB-withdrawal in the presence of DMSO or GSK-J4, for the durations indicated. **(B)** Gene expression was compared between MCF10A cells treated with either vehicle or TGFB for 6 days. **(C)** Gene expression was compared between MCF10A cells treated with TGFB for 6 days, and cells treated with TGFB for 6 days, followed by TGFB-withdrawal for 10 days. **(D)** Gene expression was compared between MCF10A cells treated with TGFB for 6 days, and subsequently subjected to TGFB-withdrawal for 10 days, in the presence of either vehicle control (DMSO), or 50 nM GSK-J4. Expression was normalized to GAPDH. Data are presented as the mean and standard deviation of triplicate experiments. P-values were calculated by unpaired t-tests and are corrected for multiple comparisons by the Holm-Sidak method. ns=not significant, *p<0.05, **p<0.01, ***p<0.001, ****p< 0.0001. **(E)** The table summarizes the expression changes for each gene across the different experimental conditions in panels B-D. Genes that exhibit significantly different expression in the direction indicated by each individual criterion are marked with “Yes”, while significant changes in the other direction or non-significant differences are marked with “No”. Genes marked with “Yes” for all three conditions are highlighted in yellow to indicate that they are suppressed following EMT, and that their re-expression following MET is sensitive to inhibition of H3K27me3-demethylase activity by GSK-J4.

In order to test whether KDM6A is involved in the resolution of bivalency and the reactivation of epithelial genes during EMT-reversal/MET, we exposed MCF10A cells to TGFB to elicit EMT, followed by TGFB-withdrawal to instigate MET. Furthermore, during TGFB-withdrawal, we treated the cells with either DMSO (as a vehicle control) or the H3K27me3-demethylase inhibitor GSK-J4 [37, 38] (Figure 5A). We confirmed the induction of EMT and MET by immunostaining for the intermediate filament protein vimentin, a well-established marker of mesenchymal cells. As expected, TGFB-induced EMT was accompanied by markedly increased immunostaining for vimentin, relative to vehicle-treated MCF10A cells, whereas TGFB-withdrawal, commensurate with MET, was evidenced by greatly diminished vimentin levels (Supplementary Figure 3). Interestingly, cells subjected to concurrent TGFB-withdrawal and GSK-J4 treatment exhibited intermediate levels of vimentin expression, suggesting that only a partial MET was achieved, consistent with a role for KDM6A activity in the restoration of the epithelial phenotype during MET progression (Supplementary Figure 3).

We next used this model to measure the mRNA expression of a subset of bivalent genes in Groups I-IV. The expression of the majority of genes in Groups I-III was consistently downregulated to a statistically significant degree following TGFB-induced EMT in MCF10A cells (Group I: 15/22, Group II: 6/8, Group III: 5/8), while the expression of the majority of genes in Group IV remained unchanged, as would be predicted by their constitutively bivalent status (Figure 5B). Modest fluctuations observed in the expression of Group IV genes are potentially due to additional gene expression regulatory mechanisms. We next examined how expression of these select bivalent genes was altered during TGFB-withdrawal/MET. In Group I, the majority of genes that were downregulated during EMT were subsequently reactivated during MET (10/15) and this pattern was also seen for Group II (5/6), Group III (5/5) and Group IV (2/2) (Figure 5C). Remarkably, the MET-associated re-expression of nearly all the genes that displayed this pattern of regulation was sensitive to inhibition of KDM6A by GSK-J4 (Group I: 9/10, Group II: 5/5, Group III: 4/5, Group IV 1/2) (Figure 5D). While sensitivity to GSK-J4 was expected for genes in Groups I and III (Figure 5E), which exhibited a gain of H3K27me3 to become bivalent in our Twist-driven model of EMT, we did not expect to observe sensitivity to GSK-J4 in Group II genes, which gained H3K4me3 to become bivalent. As expected, the expression of Group IV genes, which retain a bivalent status throughout EMT and MET, remained largely unaltered, consistent with the notion that the establishment of bivalency at these loci is independent of KDM6A. Collectively, our data demonstrate that KDM6A-dependent H3K27me3 demethylation plays an important role in the re-expression of bivalent genes, involved in proliferation, differentiation and cell adhesion, during MET.

### Expression of KDM6A and its target genes, which become bivalent during EMT, is lower in CSC-enriched, triple-negative breast cancers

**Figure 6:**
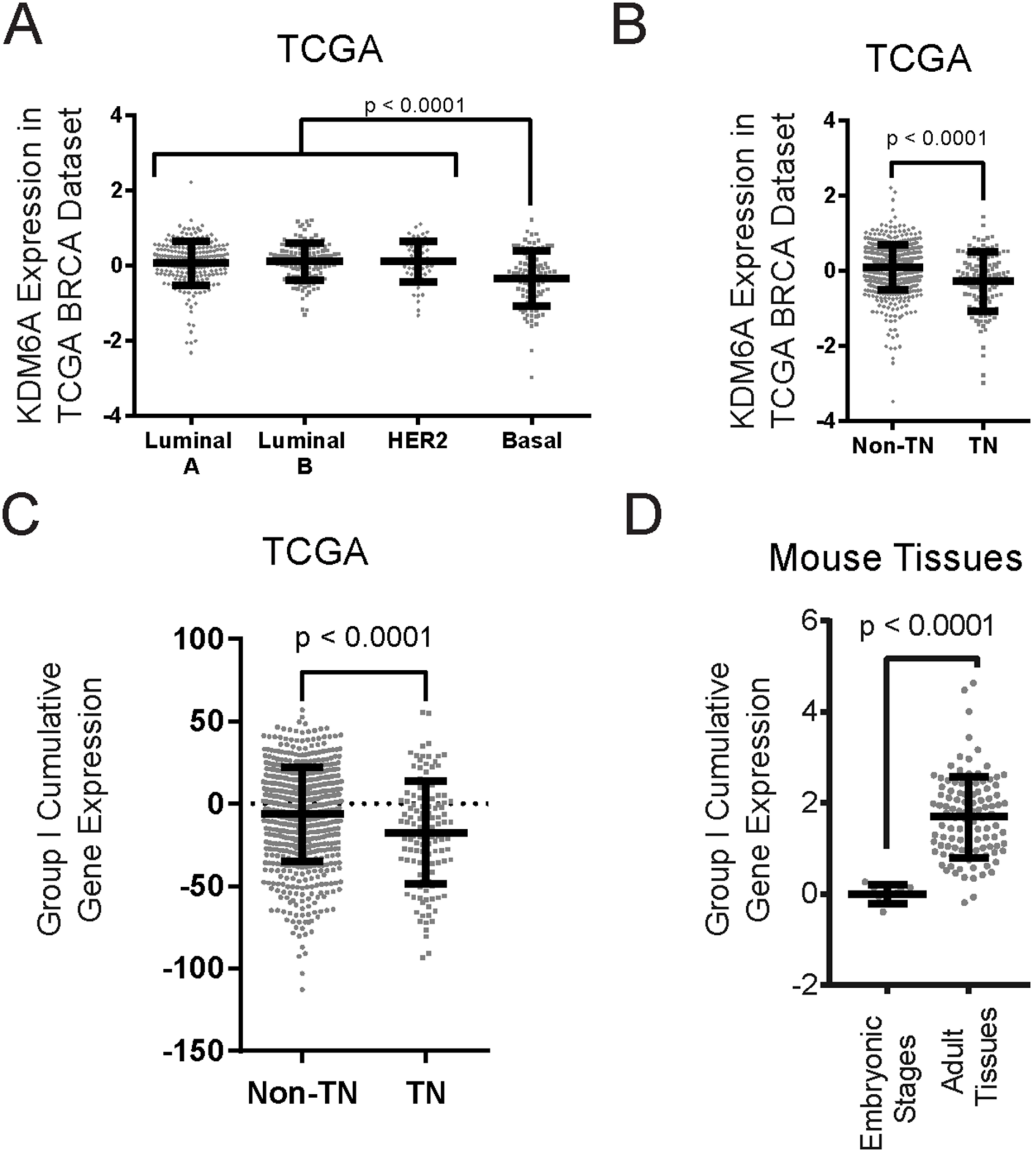
KDM6A and Group I bivalent genes are expressed at lower levels in basal-like and triple-negative breast cancers Breast cancers from the TCGA datasets were categorized based on PAM50 classification **(A)** or ER-, PR- and HER2-status **(B)**, and the expression of KDM6A was deduced from the available RNA-Seq data. P-values were calculated using Student’s two-tailed t-test. **(C)** The cumulative expression levels of all the Group I genes were compared between breast cancer samples, classified as triple-negative (TN) or non-triple negative (Non-TN). P-values were calculated using Student’s two-tailed t-test. **(D)** The cumulative expression levels of orthologous Group I genes from embryonic and adult mouse tissue samples were compared and plotted. Each dot represents a different timepoint during embryo development (Embryonic Stages) or tissue of origin (Adult Tissues). P-values were calculated using Student’s two-tailed t-test.

Since we observed reduced KDM6A expression levels in cells that have undergone EMT and in stem cell-rich populations, we next sought to determine if breast cancer subtypes, enriched for EMT/CSC properties, also exhibit lower KDM6A levels. Breast cancers are markedly heterogeneous and can be classified into distinct subsets on the basis of their gene expression profiles or histopathological criteria. Differential gene expression profiling separates breast cancers into “intrinsic” molecular subtypes designated luminal A, luminal B, HER2-enriched and basal-like [39], which includes the majority of the recently characterized claudin-low tumors [40]. Of these, the basal-like tumors are most enriched for mesenchymal and CSC features [41, 42]. Consistent with these characteristics, the basal-like tumors in The Cancer Genome Atlas (TCGA) dataset express lower KDM6A levels compared to the other subtypes examined (Figure 6A).

Based on immunohistochemical criteria—more often used clinically—breast cancers can be broadly classified into hormone-receptor positive luminal, HER2-expressing, and triple-negative subtypes. Triple-negative breast cancers (TNBCs), i.e. tumors lacking ER-, PR- or HER2-expression, are also enriched for EMT/CSC features when compared to ER-, PR- or HER2-positive breast cancers, consistent with the fact that the majority of basal tumors are also TNBCs [39]. Importantly, KMD6A expression is lower in TNBCs compared to non-TNBCs (Figure 6B). Furthermore, we determined that the cumulative expression of the Group I genes, which become bivalent following EMT through addition/retention of H3K27me3, is also decreased in TNBCs compared to non-TNBCs (Figure 6C). Moreover, these Group I genes are suppressed in the embryonic stages of mouse development relative to their expression in a spectrum of adult/differentiated mouse tissues (Figure 6D). These data support our model that KDM6A expression/activity is a key enforcer of the epithelial phenotype and that loss of KDM6A expression correlates with EMT and the acquisition of stem-like properties during development and cancer progression.

## DISCUSSION

**Figure 7:**
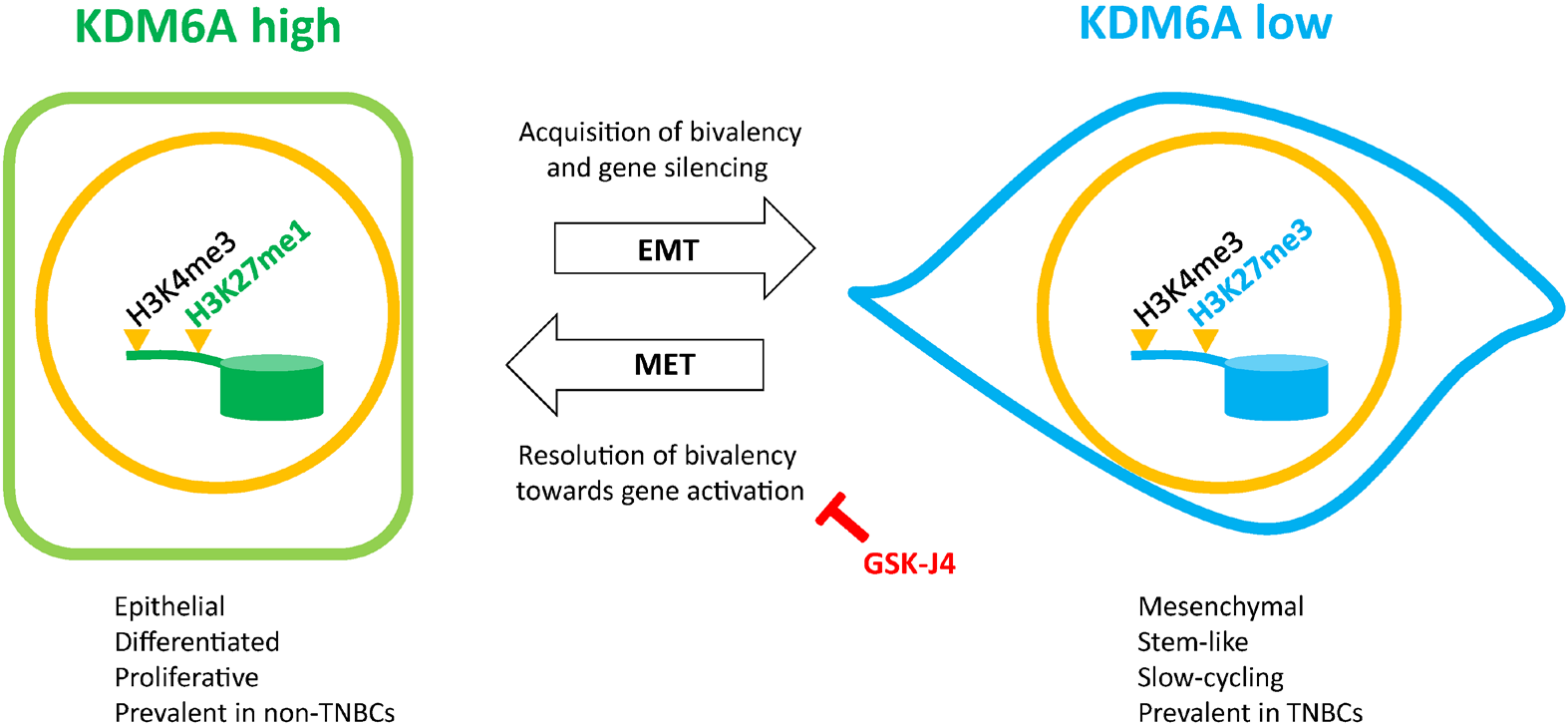
Schematic representation of the regulation of bivalency by KDM6A during EMT/MET In mammary epithelial cells, KDM6A constitutively catalyzes the removal of di- and trimethyl groups from the H3K27me3 repressive mark of bivalent promoters (H3K4me3/H3K27me3), thus unmasking an active configuration (H3K4me3/H3K27me1). KDM6A target genes, associated with differentiation, proliferation and intercellular adhesion, are hence maintained in a transcriptionally active state reinforcing the epithelial phenotype. Following EMT and loss of KDM6A expression, cells accumulate H3K27me3 at KDM6A target promoters leading to the transcriptional repression of genes associated with differentiation, proliferation and intercellular adhesion, and to the acquisition of a mesenchymal/stem-like/slow-cycling phenotype. Conversely, KDM6A levels are restored during MET, concomitant with target gene activation/resolution of bivalency through H3K27me3 removal, and restoration of epithelial attributes. Inhibition of KDM6A, using the H3K27me3-demethylase inhibitor GSK-J4, prevents the re-expression of bivalent genes during MET. Non-TNBCs and TNBCs are enriched for cells in the “KDM6A high” and “KDM6A low” states respectively.

The EMT and MET processes have been increasingly recognized as critical cellular reprogramming events facilitating carcinoma progression and metastasis [1–3]. The induction of EMT endows stationary carcinoma cells with the migratory and invasive potential, as well as the stemness attributes, required to navigate the sequential steps of the metastatic cascade and initiate micrometastases [4, 43–45]. Upon encountering the foreign microenvironment at the distant site, disseminated tumor cells undergo MET and revert to an epithelial-like proliferative phenotype, which is important for colonization and formation of overt epithelial metastatic outgrowths [6, 7, 46–49]. While key molecular effectors of the EMT/MET program have been deciphered, the changes in the chromatin architecture underlying EMT/MET, and the epigenetic regulators effecting these changes, are only just beginning to be explored. Herein, we identify increased H3K27me3 occupancy as the predominant modification leading to the establishment of bivalent domains and gene silencing following EMT. Most importantly, we establish a role for the H3K27me3-demethylase KDM6A in the resolution and activation of bivalent genes accompanying MET and the transition between the mesenchymal and epithelial states (Figure 7).

We previously reported a significantly increased incidence of bivalent genes in epithelial cells induced to undergo EMT via ectopic expression of Twist, relative to their vector-transduced epithelial counterparts [20]. We made the striking observation that 47% of the bivalent domains, established *de novo* during EMT, arise through the accumulation of H3K27me3 at monovalent premarked H3K4me3-promoters [20]. Through gene ontology analysis, we found herein that Group I—encompassing H3K4me3-premarked genes that accrue H3K27me3 following EMT—likely represents a coordinately-regulated EMT/MET-associated structural gene module that controls cellular architecture, inter-cellular adhesion and intrinsic motility, in addition to a gene module involved in the regulation of proliferation. Accordingly, we found that Group I genes are most markedly differentially expressed between the epithelial and mesenchymal states and, overall, tend towards lower expression in HMLE-Twist cells compared to HMLE-vector cells, consistent with the establishment of a repressed/bivalent configuration. With these considerations in mind, we focused on the modulation of H3K27me3 as a major factor regulating the formation of bivalent domains and the silencing of gene expression following EMT.

We reasoned that the observed accumulation of H3K27me3 marks following EMT might be attributed to an increased deposition of methyl groups, mediated by the PRC2 complex, and/or to a reduction in H3K27me3-demethylase activity. However, we found that the protein levels of both EZH2 and RING1A, two key components of the PRC2 complex [33], were downregulated during TGFB-induced EMT in MCF10A cells, suggesting that H3K27me3 accumulation is not primarily achieved through increased deposition of methyl groups. We also examined the expression of the two known H3K27me3-demethylases, KDM6A and KDM6B, and found that the induction of EMT is accompanied by markedly decreased KDM6A protein levels and, conversely, by elevated KDM6B expression. As the establishment of bivalency requires the accumulation of H3K27me3 marks, we conclude that the attenuation of KDM6A-associated H3K27me3-demethylase activity is the principal mechanism leading to increased H3K27me3-occupancy at H3K4me3-premarked promoters, underpinning the emergence of newly-formed bivalent domains following EMT.

The downregulation of *CDH1*/E-cadherin expression is considered a hallmark of EMT, and the enrichment of H3K27me3 at the *CDH1* promoter has been shown to be a key factor—amongst other gene-repressive mechanisms—in silencing *CDH1* expression in many tumor contexts [50–54]. Given that H3K27me3 occupancy can be modulated through enzymatic removal, we aimed to understand how EMT-associated *CDH1* repression may be reversed during MET. First, using MCF10A cells, we showed that, while KDM6A protein levels decline during TGFB-induced EMT, its expression is restored following TGFB-withdrawal and EMT-reversal/MET. Our findings, herein, clearly show a progressive accumulation of H3K27me3 at the *CDH1* promoter during TGFB-induced EMT, which correlates with a marked reduction in *CDH1* transcript levels. Following TGFB-withdrawal and MET, the H3K27me3 mark is rapidly lost from the *CDH1* promoter, preceding the upregulation of E-cadherin expression and restoration of the epithelial phenotype. These results are consistent with the notion that KDM6A enhances *CDH1* transcription during MET, by demethylating the repressive H3K27me3 mark previously deposited at this locus during EMT. This is also supported by the recent observation that KDM6A is necessary for H3K27me3 demethylation at the *CDH1* locus in colon cancer cells [55]. Our results further suggest that KDM6A is constitutively present at the *CDH1* locus in epithelial cells, thus regulating the basal levels of E-cadherin. Conversely, the KDM6A occupancy at the *CDH1* promoter is diminished in mesenchymal cells and is inversely correlated with H3K27me3 levels.

We also aimed to understand how EMT-associated bivalent gene repression may be reversed during MET. Using the H3K27me3-demethylase inhibitor, GSK-J4, in our dynamic MCF10A EMT/MET model, we demonstrated that KDM6A plays a critical role in the MET-associated resolution and reactivation of bivalent genes. Significantly, our results indicate that GSK-J4 treatment, concurrent with TGFB-withdrawal/MET, prevents the activation of numerous bivalent genes implicated in proliferation, differentiation and cell-cell adhesion. Notably, bivalent genes that are repressed during EMT and de-repressed following MET in a GSK-J4 sensitive manner, include genes encoding regulators of cell growth and differentiation (*CBX4, CCNA1, CDK4, FAM60A*), components of desmosomes or microtubules functioning in the regulation of cell adhesion, polarity and migration (*PKP2*), and members of signal transduction pathways (*FZD8, MAP3K9*). Similarly, bivalent genes have been shown to be resolved by KDM6A during mouse ES cell differentiation [27]. Furthermore, KDM6A-demethylase activity has been shown to regulate the reprogramming that underpins the derivation of induced pluripotent stem cells from somatic cells [56], a process wherein MET is considered a critical initiating event [57, 58]. Overall, these results suggest an important role for KDM6A in the reactivation of bivalent genes, associated with the establishment of a proliferative, differentiated, epithelial phenotype following the induction of MET.

Consistent with published reports, we found that KDM6B protein expression was increased during TGFB-induced EMT [26] and decreased following MET, in a manner inversely correlated with the levels of KDM6A. Since both KDM6A and KDM6B act by removing repressive H3K27me3 marks, and, as they exhibit differential expression patterns in the distinct settings of EMT and MET, it is unlikely that they target the same genes. This is supported by the inability of either demethylase to compensate for the loss of the other during embryonic development, which implies significant non-overlap in either their respective target genes, their tissue-specific expression patterns, or both [59]. Importantly, although GSK-J4-mediated inhibition of KDM6B is likely to lead to the accumulation of H3K27me3 marks in a distinct set of target genes, the fact that KDM6B levels are decreased during TGFB-withdrawal/MET renders GSK-J4 inhibition of KDM6B redundant in respect to blocking the reactivation of bivalent genes during MET.

Remarkably, KDM6A protein levels are significantly decreased in the stem-like subpopulations of established mammary cell lines, compared to non-stem cell subsets, consistent with the links between stem-like attributes and the EMT phenotype [4, 5]. Taken together with the finding that the expression of KDM6A is restored in cells undergoing EMT-reversal/MET, we propose that KDM6A target gene occupancy is mostly associated with differentiation, and markedly diminished in the mesenchymal, stem-like state. This expression pattern is in concert with many recent findings, including an unbiased genetic screen that identified KDM6A as an EMT-suppressor in hepatocellular carcinoma cells [60–63]. In the present study, we also found that many Group I genes, rendered bivalent and silenced through retention of the H3K27me3 mark in the absence of KDM6A, are functionally involved in proliferation. Consistent with this, a recent study linked the high expression of KDM6A to the proliferative capacity of breast cancer cell lines [62]. Taken together with the attenuated levels of KDM6A in stem-like cells, we suggest that low overall KDM6A expression and/or activity may be a contributing factor in the establishment of the relative quiescence manifested by normal and cancer stem cells [64, 65]. Moreover, we found that the expression of KMD6A, and its Group I target genes, is lower in CSC-enriched TNBCs compared to non-TNBCs, lending support to the potential clinical relevance of our findings.

Since tumor cells can disseminate early during tumor progression, the initial steps of metastatic dissemination, facilitated by EMT, may not represent amenable therapeutic targets for tumors with a propensity to metastasize. However, the reliance of colonization on the induction of MET at the distant sites may provide a potential therapeutic window for intervention. Our findings, that KDM6A activity is harnessed towards the resolution of bivalency and subsequent gene activation during MET, suggest that inhibiting KDM6A activity may be a viable therapeutic strategy to compromise the onset of MET-associated proliferation, differentiation, and colonization. Indeed, the small molecule inhibitor of H3K27me3-demethylase activity, GSK-J4, represents a promising drug candidate in this regard [66]. In keeping with our finding that KDM6A maintains the expression of E-cadherin, a recent publication by Choi *et al.* [60] highlighted a role for KDM6A in suppressing the expression of the genes encoding the EMT-inducing transcription factors SNAIL, ZEB1, and ZEB2, thereby acting essentially as an EMT-suppressor. Intriguingly, however, this study showed that KDM6A elicits the repression of *SNAIL*, *ZEB1*, and *ZEB2* in an H3K27me3-demethylase-independent manner, through its function as a scaffold protein, capable of recruiting LSD1, HDAC1, and DNMTs to promoters bound by MLL4 [60]. Taken together, these findings present a compelling case for the development of small molecules that selectively block the KDM6A H3K27me3-demethylase activity, and/or modulate its scaffold function. We suggest that such molecules may comprise promising therapeutic candidates to suppress EMT or prevent MET-associated colonization by maintaining disseminated tumor cells in a dormant/quiescent state.

Overall, we demonstrate that KDM6A expression is decreased following EMT, and suppressed in stem-like subpopulations and TNBCs, with the corollary that genes involved in proliferation, cell adhesion, and differentiation are repressed through *de novo* deposition and/or retention of the H3K27me3 repressive mark. Significantly, KDM6A plays a critical role in the MET-associated resolution and reactivation of bivalent genes by removing H3K27me3 marks deposited during EMT. Taken together with a direct role for KDM6A in the maintenance of basal E-cadherin levels, these results suggest that KDM6A is a critical enforcer of the epithelial phenotype. These observations are consistent with a role for KDM6A in the resolution and activation of numerous bivalent genes, involved in proliferation and differentiation, to facilitate colonization during the latter stages of metastasis.

## MATERIALS AND METHODS

### Cell culture and drug treatments

Immortalized human mammary epithelial cells (HMLE), including cells expressing the empty vector (pWZL), Snail or Twist were maintained as in Mani *et. al* [4]. MCF10A cells were cultured in 2D as a monolayer, as described in Debnath *et al.* [67]. 4T1 cells were cultured as in Yang *et al.* [68]. MCF7 and MDA-MB-231 cell lines were cultured in Dulbecco’s Modified Eagle Medium (DMEM) with 10% fetal bovine serum and penicillin/streptomycin. TGFB (R&D Biosystems, Minneapolis, MN, USA) was reconstituted in sterile 4 mM HCl containing 0.1% bovine serum albumin. TGFB was added to the culture medium at a concentration of 2.5 ng/ml unless otherwise indicated, with the reconstitution solution serving as a vehicle control. GSK-J4 (Selleckchem, Houston, TX, USA) was added to the culture medium at a concentration of 50 nM, with DMSO as a vehicle control.

### Antibodies, immunoblotting, and immunofluorescence

Primary antibodies, used for immunoblotting and immunofluorescence, were raised against the following antigens: KDM6A (A302-374A; Bethyl Laboratories, Montgomery, TX, USA, and A302-374A; NBP1-80628, Novus Biologicals, Danvers, MA, USA), vimentin (10082-248; Proteintech, Rosemont, IL, USA), N-cadherin (D4R1H; Cell Signaling Technology, Danvers, MA, USA), E-cadherin (61081; BD Biosciences, San Jose, CA, USA), COX IV (926-42214; Licor, Lincoln, NE, USA), KDM6B (NBP1-06640; Novus Biologicals), EZH2 (5246; Cell Signaling, Danvers, MA, USA), RING1A (13069; Cell Signaling) and β-actin (ab8227; Abcam, Cambridge, MA, USA).

For immunoblotting, proteins were extracted by lysing cells in ice-cold radio-immunoprecipitation (RIPA) buffer containing protease and phosphatase inhibitors (Roche, Nutley, NJ, USA). Protein was quantified using the Bradford Assay (BioRad, Hercules, California, USA). Cell lysates (50 μg) were resolved using SDS-PAGE and transferred to polyvinylidene difluoride (PVDF) membranes. Membranes were probed with primary antibodies overnight at 4°C, followed by extensive washing with TBST, and incubation with secondary antibodies for 1 hr at room temperature. Chemiluminescent signals were detected with ECL™ prime (Thermo Fisher Scientific, Waltham, MA USA) using the Biorad ChemiDoc system.

Immunofluorescent staining was conducted as described in Mani *et. al.* [4], and images were acquired using an inverted Zeiss Axio Observer fluorescent microscope or an inverted Nikon Eclipse Ts2R fluorescent microscope.

### PKH26 labeling

PKH26 (Sigma-Aldrich, St. Louis, MO, USA) labeling was performed according to the manufacturer’s protocol. Label-retaining cells were sorted by FACS during which non-viable cells were excluded by propidium iodide staining. Sorted cells were then fixed in suspension for 10 minutes in 10% paraformaldehyde. Equal numbers of PKH26^-ve/low^ and PKH26^+ve^ cells were centrifuged onto slides by spinning for 2 minutes at 1500 rpm in a Cytospin 4 Cytocentrifuge (Thermo Fisher Scientific), or pelleted and resuspended in growth media and allowed to attach to coverslips. Cytospun cells were subsequently immunostained as described in Mani *et. al.* [4]. Images were acquired using a Cytation™ 3 Cell Imaging Multi-Mode Reader (BioTek, Winooski, VT, USA), and analyzed for fluorescence intensity using Gen5 image analysis software (BioTek).

### Quantitative reverse-transcription PCR and chromatin immunoprecipitation

Total RNA was isolated using Trizol (Ambion, Foster City, CA, USA) according to the manufacturer’s instructions. Relative quantification of the mRNA levels was performed using the comparative Ct method with glyceraldehyde 3-phosphate dehydrogenase (*GAPDH*) as the reference gene and with the formula 2^−ΔΔCt^. All quantitative reverse transcription-PCR (RT-PCR) experiments were run in triplicate and a mean value was used for the determination of mRNA levels.

To select bivalent genes for follow-up RT-PCR analysis, as presented in Figure 5, we first narrowed the sets of bivalent genes to those with appreciable levels of expression [Fragments Per Kilobase of transcript per Million mapped reads (FPKM)>10] in our RNA-Seq-derived dataset of gene expression in epithelial HMLE-vector cells. Next, we randomly selected 25 genes from Group I, and 10 each from Groups II, III and IV, and designed RT-PCR primers (Supplementary Table 2). Only those genes that were detectable by RT-PCR are presented in Figure 5.

Chromatin immunoprecipitation, followed by quantitative PCR analysis, was performed as described in Malouf *et al.* [20].

### Statistical analyses of gene expression, gene ontology, and gene set overlap

RNA-Seq expression data were extracted from The Cancer Genome Atlas (TCGA) for breast cancer patients (BRCA). We categorized samples by PAM50 classification or by ER, PR and HER2 status. Samples with definitively negative values for ER, PR and HER2 were categorized as triple-negative (TN), while samples with a definitively positive value for ER, PR or HER2 were categorized as non-triple negative (Non-TN). We compared gene expression levels using a two-sided t-Test with unequal variance. The mouse tissue gene expression data were derived from Soundararajan *et al.* [69]. Gene ontology analysis was performed by uploading selected gene name sets to the GOrilla web server [70] as the target set, using all of the gene IDs, from our previously reported ChIP-Seq data, as background [20]. The association between two sets of genes was examined using Fisher’s exact test to find statistical significance. A small p-value means that the association (or overlap) is significant. Odds ratio was employed to measure the strength of association. Generally, an odds ratio greater than one indicates a strong association.

## SUPPLEMENTARY MATERIALS FIGURES AND TABLES




