# Supplementary material for: The H3K27me3-demethylase KDM6A is suppressed in breast cancer stem-like cells, and enables the resolution of bivalency during the mesenchymal-epithelial transition
Source: Oncotarget. 2017 Jul 10;8(39):65548–65. doi: 10.18632/oncotarget.19214 (PMC5630352; doi:10.18632/oncotarget.19214)
Supplement: Supplementary file 1 [file oncotarget-08-65548-s001.pdf]

The H3K27me3-demethylase KDM6A is suppressed in breast cancer stem-like cells, and enables the resolution of bivalency during the mesenchymal-epithelial transition

SUPPLEMENTARY MATERIALS

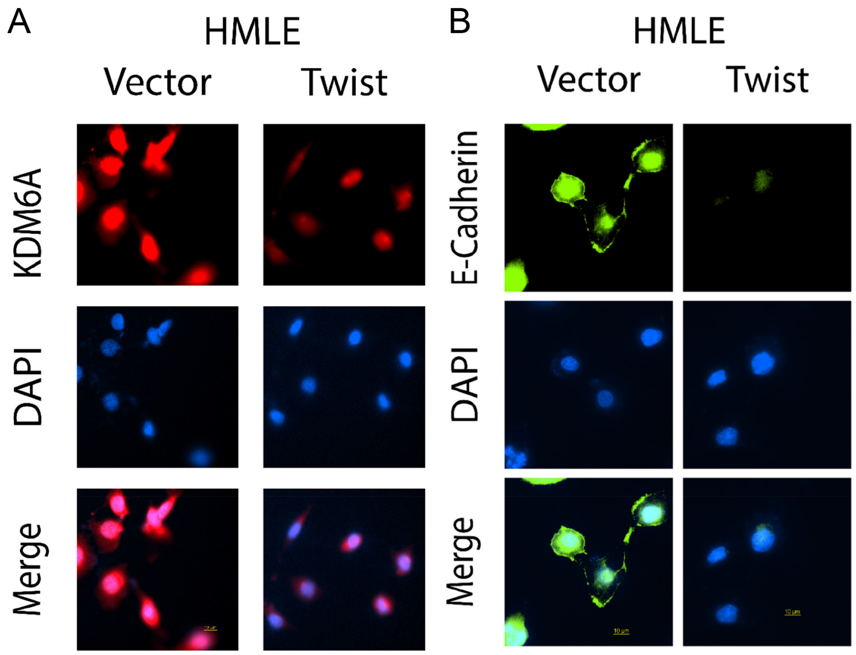

Supplementary Figure 1: HMLE-vector and HMLE-Twist cells were fixed and immunostained for **(A)** KDM6A (red) or **(B)** E-cadherin (green). Nuclei were counterstained with DAPI (blue). Scale bars, 10 µm.

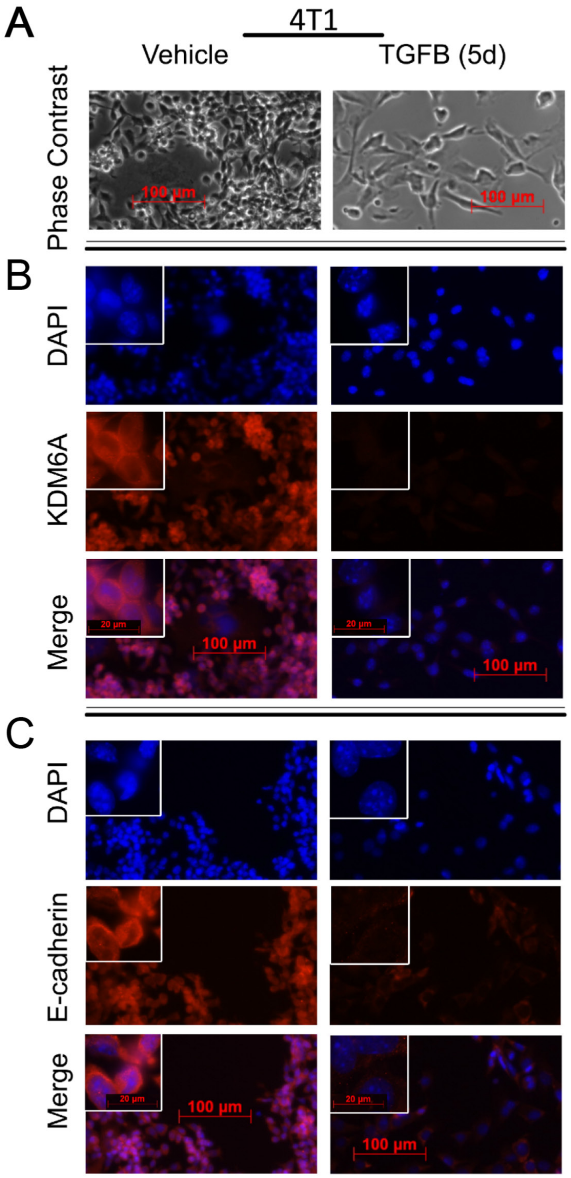

Supplementary Figure 2: KDM6A protein levels are decreased following TGFB-induced EMT in 4T1 cells. 4T1 cells were cultured in 2D in the presence of either a vehicle control (sterile 4 mM HCl containing 0.1% bovine serum albumin) or TGFB (5 ng/ml) for 5 days. **(A)** Cell morphology was imaged by phase-contrast microscopy (top panels). **(B, C)** KDM6A (B) or E-cadherin (C) expression and subcellular localization (red) were assessed by immunofluorescent staining with the respective antibodies. Nuclei were counterstained with DAPI (blue). Scale bars, 100 µm. The insets represent magnified images of selected areas. Scale bars, 20 µm.

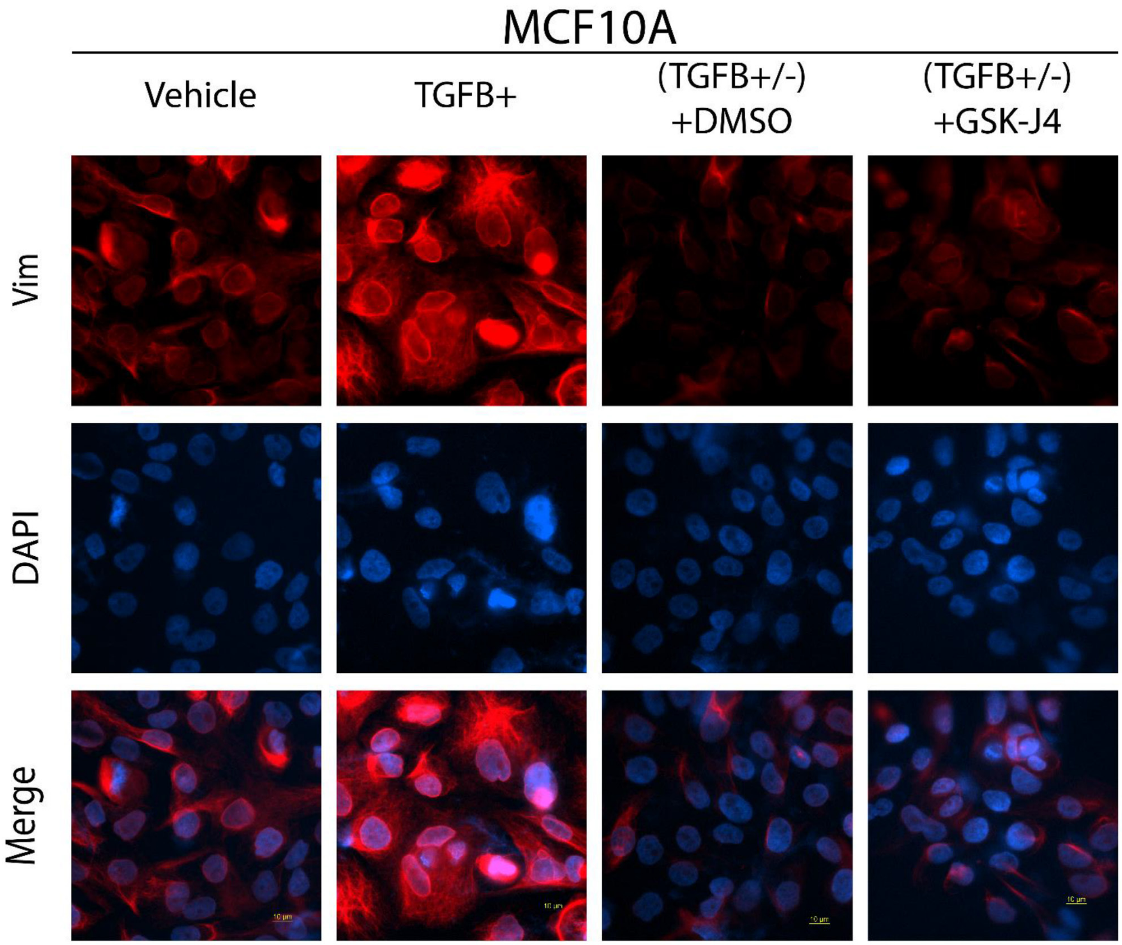

Supplementary Figure 3: Vimentin immunostaining is increased following EMT and decreased following MET in MCF10A cells. MCF10A cells were treated with vehicle or TGFB for 6 days prior to fixation. In parallel, MCF10A cells exposed to TGFB for 6 days, to induce EMT, were subsequently subjected to TGFB-withdrawal in the presence of either vehicle (DMSO) or GSK-J4 for a further 10 days. Cells were fixed and immunostained for vimentin (Vim; red). Nuclei were counterstained with DAPI (blue). TGFB+ denotes addition of TGFB for 6 days. TGB+/- signifies addition of TGFB for 6 days, followed by TGFB-withdrawal for a further 10 days. The addition of either vehicle (DMSO) or GSK-J4 is indicated. Scale bars, 10 µm.

Supplementary Table 1: H3K4me3 and H3K27me3 status of promoters in epithelial HMLE-vector cells and mesenchymal HMLE-Twist cells

See Supplementary File 1

Supplementary Table 2: Primer sequences used in quantitative RT-PCR or ChIP

See Supplementary File 1
