# Supplementary material for: The H3K27me3-demethylase KDM6A is suppressed in breast cancer stem-like cells, and enables the resolution of bivalency during the mesenchymal-epithelial transition
Source: Oncotarget. 2017 Jul 10;8(39):65548–65. doi: 10.18632/oncotarget.19214 (PMC5630352; doi:10.18632/oncotarget.19214)
Supplement: Supplementary file 2 [file oncotarget-08-65548-s002.docx]

**Supplementary Table 1: H3K4me3 and H3K27me3 status of promoters in epithelial HMLE-vector cells and mesenchymal HMLE-Twist cells**

| **Gene name** | **Gene_id** | **Gene position** | **K27me3 in HMLE-vector?** | **K4me3 in HMLE-vector?** | **K27me3 in HMLE-Twist?** | **K4me3 in HMLE-Twist?** | **Bivalent in HMLE-vector?** | **Bivalent in HMLE-Twist?** |
| --- | --- | --- | --- | --- | --- | --- | --- | --- |
| WDR8 |  | chr1:3537191-3556531 | N | Y | Y | Y | No | Yes |
| TMEM56 |  | chr1:95355481-95435747 | N | Y | Y | Y | No | Yes |
| CAMK2N2 | NM_001098202 | chr3:185459696-185461945 | N | Y | Y | Y | No | Yes |
| RABL4 |  | chr22:35484200-35502060 | N | Y | Y | Y | No | Yes |
| FAM119B | NM_023011 | chr12:56452649-56462591 | N | Y | Y | Y | No | Yes |
| DEPDC6 | NM_001114937 | chr8:120955080-121132338 | N | Y | Y | Y | No | Yes |
| C10orf41 | NM_001136127 | chr10:76831291-76838745 | N | Y | Y | Y | No | Yes |
| ABI1 | NM_000673 | chr10:27075530-27189965 | N | Y | Y | Y | No | Yes |
| C9orf122 | NM_000213 | chr9:38611084-38613275 | N | Y | Y | Y | No | Yes |
| PLAGL1 |  | chr6:144303129-144427428 | N | Y | Y | Y | No | Yes |
| DPH1 | NM_020468 | chr17:1880180-1893475 | N | Y | Y | Y | No | Yes |
| LOC440926 |  | chr1:224317050-224326326 | N | Y | Y | Y | No | Yes |
| MARCH9 | NM_001788 | chr12:56435166-56439956 | N | Y | Y | Y | No | Yes |
| MIR1181 |  | chr19:10375133-10375214 | N | Y | Y | Y | No | Yes |
| SNORD15A |  | chr11:74789082-74789230 | N | Y | Y | Y | No | Yes |
| RPS3 |  | chr11:74788209-74794381 | N | Y | Y | Y | No | Yes |
| ARPC2 | NM_001099402 | chr2:218790364-218827316 | N | Y | Y | Y | No | Yes |
| MYL12A |  | chr18:3237527-3246234 | N | Y | Y | Y | No | Yes |
| ATP1A1 | NM_001098533 | chr1:116717317-116748917 | N | Y | Y | Y | No | Yes |
| TGFBI |  | chr5:135392482-135427406 | N | Y | Y | Y | No | Yes |
| RPN2 |  | chr20:35240869-35303437 | N | Y | Y | Y | No | Yes |
| DEK | NM_001093770 | chr6:18332380-18372778 | N | Y | Y | Y | No | Yes |
| RAD21 |  | chr8:117927354-117956286 | N | Y | Y | Y | No | Yes |
| VDAC1 |  | chr5:133335505-133368332 | N | Y | Y | Y | No | Yes |
| GJB2 |  | chr13:19659605-19665114 | N | Y | Y | Y | No | Yes |
| OAT |  | chr10:126075861-126097535 | N | Y | Y | Y | No | Yes |
| UQCRFS1 |  | chr19:34390006-34395976 | N | Y | Y | Y | No | Yes |
| ETFA | NM_021253 | chr15:74295683-74390865 | N | Y | Y | Y | No | Yes |
| PSMD1 |  | chr2:231629852-231745717 | N | Y | Y | Y | No | Yes |
| NPM3 |  | chr10:103531072-103533160 | N | Y | Y | Y | No | Yes |
| CDKN3 | NM_001040113 | chr14:53933422-53956682 | N | Y | Y | Y | No | Yes |
| COX7A2L | NM_001177355 | chr2:42431147-42441860 | N | Y | Y | Y | No | Yes |
| TNFRSF12A |  | chr16:3010313-3012384 | N | Y | Y | Y | No | Yes |
| PERP |  | chr6:138451335-138470353 | N | Y | Y | Y | No | Yes |
| APLP2 | NM_007293 | chr11:129444925-129519915 | N | Y | Y | Y | No | Yes |
| DSG2 | NM_020398 | chr18:27332024-27382812 | N | Y | Y | Y | No | Yes |
| CREG1 | NM_002482 | chr1:165776874-165789680 | N | Y | Y | Y | No | Yes |
| PEA15 |  | chr1:158441748-158451784 | N | Y | Y | Y | No | Yes |
| SLC38A1 |  | chr12:44863107-44949475 | N | Y | Y | Y | No | Yes |
| ARF3 | NM_001167674 | chr12:47616258-47637519 | N | Y | Y | Y | No | Yes |
| FAM91A1 | NM_001143938 | chr8:124850062-124896871 | N | Y | Y | Y | No | Yes |
| MCM6 |  | chr2:136313665-136350481 | N | Y | Y | Y | No | Yes |
| SFT2D1 |  | chr6:166653505-166675981 | N | Y | Y | Y | No | Yes |
| IGFBP7 |  | chr4:57592000-57671296 | N | Y | Y | Y | No | Yes |
| CKAP4 | NM_001143996 | chr12:105155789-105165843 | N | Y | Y | Y | No | Yes |
| H3F3A |  | chr1:224317043-224326326 | N | Y | Y | Y | No | Yes |
| FKBP4 |  | chr12:2774368-2784846 | N | Y | Y | Y | No | Yes |
| TMED3 |  | chr15:77390545-77402244 | N | Y | Y | Y | No | Yes |
| EPCAM | NM_001131010 | chr2:47449790-47467669 | N | Y | Y | Y | No | Yes |
| DSC3 | NM_001098512 | chr18:26824049-26876779 | N | Y | Y | Y | No | Yes |
| PARP1 |  | chr1:224615014-224662424 | N | Y | Y | Y | No | Yes |
| EXT1 | NM_001017392 | chr8:118880782-119193239 | N | Y | Y | Y | No | Yes |
| DSP | NM_001030288 | chr6:7486868-7531945 | N | Y | Y | Y | No | Yes |
| ANP32B | NM_001136494 | chr9:99785309-99818045 | N | Y | Y | Y | No | Yes |
| ATP6V1C1 | NM_001144904 | chr8:104102423-104154461 | N | Y | Y | Y | No | Yes |
| C8orf59 | NM_001100390 | chr8:86313539-86319895 | N | Y | Y | Y | No | Yes |
| CTNNBL1 | NM_002836 | chr20:35755847-35933934 | N | Y | Y | Y | No | Yes |
| PFDN4 |  | chr20:52257908-52269899 | N | Y | Y | Y | No | Yes |
| SLC31A2 |  | chr9:114953058-114966243 | N | Y | Y | Y | No | Yes |
| GPM6B |  | chrX:13702017-13866752 | N | Y | Y | Y | No | Yes |
| BLOC1S2 | NM_001142936 | chr10:102023702-102036104 | N | Y | Y | Y | No | Yes |
| RNF145 |  | chr5:158516996-158567412 | N | Y | Y | Y | No | Yes |
| ZFR |  | chr5:32390212-32480601 | N | Y | Y | Y | No | Yes |
| SLC35B2 |  | chr6:44329815-44333261 | N | Y | Y | Y | No | Yes |
| IWS1 |  | chr2:127954852-128000557 | N | Y | Y | Y | No | Yes |
| PAWR |  | chr12:78509875-78608921 | N | Y | Y | Y | No | Yes |
| ALDH9A1 | NM_001170794 | chr1:163898072-163934524 | N | Y | Y | Y | No | Yes |
| AKAP12 | NM_001170714 | chr6:151602826-151721385 | N | Y | Y | Y | No | Yes |
| FRG1 |  | chr4:191098967-191121353 | N | Y | Y | Y | No | Yes |
| FAM60A | NM_001136046 | chr12:31324793-31370388 | N | Y | Y | Y | No | Yes |
| CDC37 | NM_178817 | chr19:10362808-10375271 | N | Y | Y | Y | No | Yes |
| KDM5B |  | chr1:200963154-201044172 | N | Y | Y | Y | No | Yes |
| TMEM9 |  | chr1:199370523-199390255 | N | Y | Y | Y | No | Yes |
| PFKM |  | chr12:46799279-46826454 | N | Y | Y | Y | No | Yes |
| STXBP3 |  | chr1:109090807-109153671 | N | Y | Y | Y | No | Yes |
| GRB2 |  | chr17:70825751-70913384 | N | Y | Y | Y | No | Yes |
| CTSL2 | NM_001136024 | chr9:98834758-98841360 | N | Y | Y | Y | No | Yes |
| FBXO3 |  | chr11:33719065-33752647 | N | Y | Y | Y | No | Yes |
| PAK1 |  | chr11:76710707-76862756 | N | Y | Y | Y | No | Yes |
| MRPL11 |  | chr11:65959125-65962886 | N | Y | Y | Y | No | Yes |
| CD47 | NM_001145966 | chr3:109244630-109292625 | N | Y | Y | Y | No | Yes |
| STK24 |  | chr13:97900455-98027397 | N | Y | Y | Y | No | Yes |
| DYM | NM_001142503 | chr18:44824169-45241077 | N | Y | Y | Y | No | Yes |
| C1orf122 | NM_001996 | chr1:38046059-38047713 | N | Y | Y | Y | No | Yes |
| F11R | NM_001139442 | chr1:159231624-159257757 | N | Y | Y | Y | No | Yes |
| PINX1 |  | chr8:10660293-10734709 | N | Y | Y | Y | No | Yes |
| UBE2E2 |  | chr3:23219787-23607300 | N | Y | Y | Y | No | Yes |
| KDELR1 |  | chr19:53577638-53586622 | N | Y | Y | Y | No | Yes |
| PKP2 |  | chr12:32834946-32941047 | N | Y | Y | Y | No | Yes |
| SRXN1 |  | chr20:575267-581890 | N | Y | Y | Y | No | Yes |
| PAK2 |  | chr3:197951124-198043915 | N | Y | Y | Y | No | Yes |
| PDXDC1 |  | chr16:14976333-15039053 | N | Y | Y | Y | No | Yes |
| FAM84B | NM_001112734 | chr8:127633868-127639648 | N | Y | Y | Y | No | Yes |
| CDS1 | NM_000260 | chr4:85723080-85791517 | N | Y | Y | Y | No | Yes |
| PGD |  | chr1:10381671-10402788 | N | Y | Y | Y | No | Yes |
| NOSIP |  | chr19:54750779-54775615 | N | Y | Y | Y | No | Yes |
| SGOL2 |  | chr2:201099109-201157063 | N | Y | Y | Y | No | Yes |
| B4GALT5 | NM_001009566 | chr20:47682889-47763828 | N | Y | Y | Y | No | Yes |
| DDX23 | NM_001130690 | chr12:47509805-47532224 | N | Y | Y | Y | No | Yes |
| PTGFRN |  | chr1:117254211-117334495 | N | Y | Y | Y | No | Yes |
| PDP1 |  | chr8:94999167-95007470 | N | Y | Y | Y | No | Yes |
| MYO1D |  | chr17:27843740-28228015 | N | Y | Y | Y | No | Yes |
| TGFA |  | chr2:70527924-70634613 | N | Y | Y | Y | No | Yes |
| METTL1 |  | chr12:56448617-56452181 | N | Y | Y | Y | No | Yes |
| PARD6G |  | chr18:76016105-76106388 | N | Y | Y | Y | No | Yes |
| HMGXB4 |  | chr22:33983444-34021799 | N | Y | Y | Y | No | Yes |
| KIAA0494 |  | chr1:46913417-46957323 | N | Y | Y | Y | No | Yes |
| FASTKD2 | NM_001136036 | chr2:207338356-207369154 | N | Y | Y | Y | No | Yes |
| SMYD5 |  | chr2:73294873-73307863 | N | Y | Y | Y | No | Yes |
| YRDC |  | chr1:38041200-38046452 | N | Y | Y | Y | No | Yes |
| MYH10 |  | chr17:8318254-8474761 | N | Y | Y | Y | No | Yes |
| IGF2BP3 |  | chr7:23316352-23476520 | N | Y | Y | Y | No | Yes |
| SLC35C2 |  | chr20:44411583-44426471 | N | Y | Y | Y | No | Yes |
| RAVER2 |  | chr1:64983365-65071500 | N | Y | Y | Y | No | Yes |
| SLC39A8 |  | chr4:103401843-103485678 | N | Y | Y | Y | No | Yes |
| SLC37A3 |  | chr7:139680020-139744780 | N | Y | Y | Y | No | Yes |
| POP4 |  | chr19:34789009-34800002 | N | Y | Y | Y | No | Yes |
| SFRP1 |  | chr8:41238633-41286147 | N | Y | Y | Y | No | Yes |
| FAM69A | NM_001032292 | chr1:93080308-93199667 | N | Y | Y | Y | No | Yes |
| FEZ1 |  | chr11:124820857-124871333 | N | Y | Y | Y | No | Yes |
| SIL1 |  | chr5:138310308-138561964 | N | Y | Y | Y | No | Yes |
| PDE8A |  | chr15:83326208-83483376 | N | Y | Y | Y | No | Yes |
| AATF | NM_004769 | chr17:32380287-32488284 | N | Y | Y | Y | No | Yes |
| EID2 | NM_001128595 | chr19:44721286-44722678 | N | Y | Y | Y | No | Yes |
| DNAJB2 | NM_001122964 | chr2:219852283-219859866 | N | Y | Y | Y | No | Yes |
| TMEM65 |  | chr8:125392339-125454121 | N | Y | Y | Y | No | Yes |
| ARFGEF2 | NM_001033952 | chr20:46971681-47086637 | N | Y | Y | Y | No | Yes |
| MORC4 |  | chrX:106070621-106130130 | N | Y | Y | Y | No | Yes |
| CCNA1 | NM_001017999 | chr13:35904408-35915019 | N | Y | Y | Y | No | Yes |
| CYP27B1 | NM_001130112 | chr12:56442383-56447243 | N | Y | Y | Y | No | Yes |
| FOXP1 |  | chr3:71329723-71715830 | N | Y | Y | Y | No | Yes |
| DHRS7B | NM_001040153 | chr17:20970849-21035428 | N | Y | Y | Y | No | Yes |
| UBE2D1 |  | chr10:59764744-59800515 | N | Y | Y | Y | No | Yes |
| C10orf58 | NM_001135917 | chr10:82158221-82182733 | N | Y | Y | Y | No | Yes |
| FRMD4B |  | chr3:69301835-69518120 | N | Y | Y | Y | No | Yes |
| TMEM188 |  | chr16:48616689-48628500 | N | Y | Y | Y | No | Yes |
| LIPG |  | chr18:45342424-45373276 | N | Y | Y | Y | No | Yes |
| PLEKHG3 |  | chr14:64240945-64280813 | N | Y | Y | Y | No | Yes |
| SNX4 |  | chr3:126648183-126721748 | N | Y | Y | Y | No | Yes |
| FAM116A | NM_005148 | chr3:57586221-57653856 | N | Y | Y | Y | No | Yes |
| RNF220 |  | chr1:44643546-44889983 | N | Y | Y | Y | No | Yes |
| MTUS1 |  | chr8:17545583-17702706 | N | Y | Y | Y | No | Yes |
| PCK2 |  | chr14:23633322-23639610 | N | Y | Y | Y | No | Yes |
| GALNTL4 |  | chr11:11248996-11600137 | N | Y | Y | Y | No | Yes |
| GINS3 |  | chr16:56983798-56997549 | N | Y | Y | Y | No | Yes |
| XPR1 |  | chr1:178867768-179126036 | N | Y | Y | Y | No | Yes |
| ALG11 | NM_001177515 | chr13:51484550-51501781 | N | Y | Y | Y | No | Yes |
| SPR |  | chr2:72968019-72972797 | N | Y | Y | Y | No | Yes |
| FAM169A | NM_005112 | chr5:74109154-74198371 | N | Y | Y | Y | No | Yes |
| TBC1D4 |  | chr13:74756809-74954251 | N | Y | Y | Y | No | Yes |
| IGSF3 |  | chr1:116918553-117011837 | N | Y | Y | Y | No | Yes |
| TP53BP2 |  | chr1:222034217-222100297 | N | Y | Y | Y | No | Yes |
| FAM120B | NM_003364 | chr6:170457768-170556162 | N | Y | Y | Y | No | Yes |
| RALGAPB |  | chr20:36534899-36640918 | N | Y | Y | Y | No | Yes |
| GIGYF2 |  | chr2:233270258-233433531 | N | Y | Y | Y | No | Yes |
| SETD7 |  | chr4:140646641-140697027 | N | Y | Y | Y | No | Yes |
| NFYB |  | chr12:103034987-103056170 | N | Y | Y | Y | No | Yes |
| ARHGEF3 | NM_001172895 | chr3:56736485-57088376 | N | Y | Y | Y | No | Yes |
| TMEM30B |  | chr14:60813841-60818283 | N | Y | Y | Y | No | Yes |
| PRDM4 |  | chr12:106650772-106679044 | N | Y | Y | Y | No | Yes |
| ALG3 | NM_001139441 | chr3:185442810-185450007 | N | Y | Y | Y | No | Yes |
| CALCOCO1 | NM_001002231 | chr12:52391169-52407574 | N | Y | Y | Y | No | Yes |
| PRRG4 |  | chr11:32808064-32832681 | N | Y | Y | Y | No | Yes |
| TBC1D7 |  | chr6:13413164-13436749 | N | Y | Y | Y | No | Yes |
| SOX4 |  | chr6:21701950-21706828 | N | Y | Y | Y | No | Yes |
| FAM8A1 | NM_175839 | chr6:17708496-17719928 | N | Y | Y | Y | No | Yes |
| POLG |  | chr15:87660539-87679030 | N | Y | Y | Y | No | Yes |
| TC2N |  | chr14:91316019-91372602 | N | Y | Y | Y | No | Yes |
| KIAA0141 |  | chr5:141283568-141301795 | N | Y | Y | Y | No | Yes |
| LPHN2 |  | chr1:82038669-82230695 | N | Y | Y | Y | No | Yes |
| C10orf28 | NM_001941 | chr10:99884370-99994644 | N | Y | Y | Y | No | Yes |
| ALDH1L2 | NM_001134432 | chr12:103937693-104002471 | N | Y | Y | Y | No | Yes |
| RRBP1 |  | chr20:17542322-17610928 | N | Y | Y | Y | No | Yes |
| ACTR5 | NM_001008218 | chr20:36810510-36834503 | N | Y | Y | Y | No | Yes |
| PRKCH |  | chr14:60858267-61087451 | N | Y | Y | Y | No | Yes |
| LRRC16A |  | chr6:25387634-25728735 | N | Y | Y | Y | No | Yes |
| KLF3 |  | chr4:38342184-38379524 | N | Y | Y | Y | No | Yes |
| VPS18 |  | chr15:38973919-38983465 | N | Y | Y | Y | No | Yes |
| RNF8 |  | chr6:37429725-37470492 | N | Y | Y | Y | No | Yes |
| ENTPD7 | NM_001142301 | chr10:101409252-101460992 | N | Y | Y | Y | No | Yes |
| SLC41A1 |  | chr1:204024843-204048784 | N | Y | Y | Y | No | Yes |
| EPB41L4B | NM_001131062 | chr9:111041832-111122842 | N | Y | Y | Y | No | Yes |
| RBM12B |  | chr8:94812903-94822400 | N | Y | Y | Y | No | Yes |
| CREM | NM_001004720 | chr10:35455806-35508781 | N | Y | Y | Y | No | Yes |
| SESN3 |  | chr11:94545780-94603894 | N | Y | Y | Y | No | Yes |
| RMND5B |  | chr5:177490633-177508085 | N | Y | Y | Y | No | Yes |
| HSPC159 |  | chr2:64534830-64542021 | N | Y | Y | Y | No | Yes |
| RRP15 |  | chr1:216525251-216577948 | N | Y | Y | Y | No | Yes |
| WWC1 |  | chr5:167651642-167831884 | N | Y | Y | Y | No | Yes |
| FAM89A | NM_001166412 | chr1:229221326-229242618 | N | Y | Y | Y | No | Yes |
| KIRREL |  | chr1:156229686-156332468 | N | Y | Y | Y | No | Yes |
| MCOLN3 |  | chr1:85256352-85286757 | N | Y | Y | Y | No | Yes |
| TMEM160 |  | chr19:52241006-52243722 | N | Y | Y | Y | No | Yes |
| TMEM55A |  | chr8:92075674-92122379 | N | Y | Y | Y | No | Yes |
| ACVR1B | NM_001137559 | chr12:50631752-50677127 | N | Y | Y | Y | No | Yes |
| RNF141 |  | chr11:10489800-10519350 | N | Y | Y | Y | No | Yes |
| IGSF8 |  | chr1:158327753-158335032 | N | Y | Y | Y | No | Yes |
| SH2D4A |  | chr8:19215766-19298008 | N | Y | Y | Y | No | Yes |
| MAP7 |  | chr6:136705564-136913485 | N | Y | Y | Y | No | Yes |
| C1orf212 | NM_006657 | chr1:35088551-35097233 | N | Y | Y | Y | No | Yes |
| NIPAL2 |  | chr8:99273562-99375797 | N | Y | Y | Y | No | Yes |
| CD83 | NM_000902 | chr6:14225843-14245127 | N | Y | Y | Y | No | Yes |
| SORL1 |  | chr11:120828170-121009681 | N | Y | Y | Y | No | Yes |
| FHOD3 |  | chr18:32131699-32614016 | N | Y | Y | Y | No | Yes |
| MAP9 |  | chr4:156483261-156517572 | N | Y | Y | Y | No | Yes |
| CITED4 | NM_002359 | chr1:41099314-41100605 | N | Y | Y | Y | No | Yes |
| ANGEL1 | NM_001164375 | chr14:76323338-76349036 | N | Y | Y | Y | No | Yes |
| TLL1 |  | chr4:167013859-167244443 | N | Y | Y | Y | No | Yes |
| TBRG1 |  | chr11:123997951-124011032 | N | Y | Y | Y | No | Yes |
| ELAVL2 | NM_000360 | chr9:23680104-23811843 | N | Y | Y | Y | No | Yes |
| TMEM67 |  | chr8:94836247-94899522 | N | Y | Y | Y | No | Yes |
| FBXL2 | NM_178450 | chr3:33293940-33403760 | N | Y | Y | Y | No | Yes |
| DKFZp761E198 | NM_007066 | chr11:65299953-65304398 | N | Y | Y | Y | No | Yes |
| ZNF697 |  | chr1:119963522-119991913 | N | Y | Y | Y | No | Yes |
| COBLL1 | NM_007169 | chr2:165249503-165406174 | N | Y | Y | Y | No | Yes |
| PCDH1 |  | chr5:141222400-141238128 | N | Y | Y | Y | No | Yes |
| SETBP1 |  | chr18:40514135-40711377 | N | Y | Y | Y | No | Yes |
| CHD6 | NM_001004060 | chr20:39464583-39680547 | N | Y | Y | Y | No | Yes |
| ELOVL7 | NM_001146016 | chr5:60083372-60175858 | N | Y | Y | Y | No | Yes |
| HOXC13 |  | chr12:52618842-52626595 | N | Y | Y | Y | No | Yes |
| LOC283392 |  | chr12:70942595-70953556 | N | Y | Y | Y | No | Yes |
| SAMD4B |  | chr19:44524947-44567377 | N | Y | Y | Y | No | Yes |
| CXADR | NM_003872 | chr21:17807200-17861137 | N | Y | Y | Y | No | Yes |
| ZNF33B |  | chr10:42404560-42453998 | N | Y | Y | Y | No | Yes |
| ARRDC2 | NM_001032999 | chr19:17972943-17985911 | N | Y | Y | Y | No | Yes |
| TRHDE |  | chr12:70952795-71345688 | N | Y | Y | Y | No | Yes |
| FZD3 |  | chr8:28407691-28477880 | N | Y | Y | Y | No | Yes |
| HTT |  | chr4:3046205-3215485 | N | Y | Y | Y | No | Yes |
| BDH1 | NM_004390 | chr3:198721050-198767255 | N | Y | Y | Y | No | Yes |
| C9orf25 | NM_016506 | chr9:34388181-34448568 | N | Y | Y | Y | No | Yes |
| JPH1 |  | chr8:75309492-75396117 | N | Y | Y | Y | No | Yes |
| GBF1 |  | chr10:103995298-104132639 | N | Y | Y | Y | No | Yes |
| SNCA |  | chr4:90864273-90977373 | N | Y | Y | Y | No | Yes |
| WASF3 |  | chr13:26029839-26161080 | N | Y | Y | Y | No | Yes |
| RASL11B |  | chr4:53423251-53427759 | N | Y | Y | Y | No | Yes |
| NEBL |  | chr10:21108910-21503122 | N | Y | Y | Y | No | Yes |
| ABHD6 | NM_000672 | chr3:58198298-58255501 | N | Y | Y | Y | No | Yes |
| MAP3K9 |  | chr14:70264606-70345641 | N | Y | Y | Y | No | Yes |
| AGPAT9 | NM_001142733 | chr4:84676676-84746050 | N | Y | Y | Y | No | Yes |
| CADM1 | NM_001159296 | chr11:114549554-114880451 | N | Y | Y | Y | No | Yes |
| ULBP2 |  | chr6:150304828-150312061 | N | Y | Y | Y | No | Yes |
| MCOLN2 |  | chr1:85163853-85235384 | N | Y | Y | Y | No | Yes |
| PIGM |  | chr1:158264085-158268407 | N | Y | Y | Y | No | Yes |
| RASEF |  | chr9:84787136-84867863 | N | Y | Y | Y | No | Yes |
| JMY |  | chr5:78567680-78658792 | N | Y | Y | Y | No | Yes |
| ELOVL4 | NM_001099274 | chr6:80681247-80714034 | N | Y | Y | Y | No | Yes |
| FAM193B | NM_001097594 | chr5:176879395-176914144 | N | Y | Y | Y | No | Yes |
| E2F8 | NM_001163940 | chr11:19202185-19219083 | N | Y | Y | Y | No | Yes |
| AARS2 | NM_001607 | chr6:44374440-44389041 | N | Y | Y | Y | No | Yes |
| MATN3 |  | chr2:20055293-20075936 | N | Y | Y | Y | No | Yes |
| MARVELD3 |  | chr16:70217570-70233369 | N | Y | Y | Y | No | Yes |
| C6orf141 | NM_001146051 | chr6:49626071-49627765 | N | Y | Y | Y | No | Yes |
| PRRG2 |  | chr19:54776398-54786077 | N | Y | Y | Y | No | Yes |
| MAPRE3 |  | chr2:27047028-27103591 | N | Y | Y | Y | No | Yes |
| PLXNA2 |  | chr1:206262210-206484288 | N | Y | Y | Y | No | Yes |
| PPP4R4 |  | chr14:93710401-93763935 | N | Y | Y | Y | No | Yes |
| FAM129A | NM_001134771 | chr1:183026788-183210305 | N | Y | Y | Y | No | Yes |
| MTMR9 |  | chr8:11179409-11223064 | N | Y | Y | Y | No | Yes |
| SOX9 |  | chr17:67628755-67634155 | N | Y | Y | Y | No | Yes |
| FAM134B | NM_016582 | chr5:16526146-16670118 | N | Y | Y | Y | No | Yes |
| RIMS2 |  | chr8:104582151-105334627 | N | Y | Y | Y | No | Yes |
| ZNF483 |  | chr9:113327267-113379945 | N | Y | Y | Y | No | Yes |
| CHD7 | NR_024434 | chr8:61753892-61942021 | N | Y | Y | Y | No | Yes |
| CTTNBP2 | NM_001099287 | chr7:117137941-117300797 | N | Y | Y | Y | No | Yes |
| EPHX4 | NM_003823 | chr1:92268120-92301681 | N | Y | Y | Y | No | Yes |
| DIS3L2 | NM_012417 | chr2:232534536-232910152 | N | Y | Y | Y | No | Yes |
| HELB |  | chr12:64982622-65018225 | N | Y | Y | Y | No | Yes |
| ZDHHC23 |  | chr3:115149437-115164517 | N | Y | Y | Y | No | Yes |
| PELI3 |  | chr11:65990911-66001384 | N | Y | Y | Y | No | Yes |
| FAM117A | NM_001143947 | chr17:45142687-45196517 | N | Y | Y | Y | No | Yes |
| HS3ST3A1 |  | chr17:13339730-13445969 | N | Y | Y | Y | No | Yes |
| ST3GAL6 |  | chr3:99934261-99995926 | N | Y | Y | Y | No | Yes |
| MARK1 |  | chr1:218768190-218904422 | N | Y | Y | Y | No | Yes |
| BHLHE41 | NM_000500 | chr12:26164227-26169270 | N | Y | Y | Y | No | Yes |
| PLXDC2 |  | chr10:20145377-20609121 | N | Y | Y | Y | No | Yes |
| DLL1 | NM_001130964 | chr6:170433218-170441622 | N | Y | Y | Y | No | Yes |
| C8orf85 | NM_001572 | chr8:118019644-118025418 | N | Y | Y | Y | No | Yes |
| ANKRD6 | NM_003796 | chr6:90199615-90400124 | N | Y | Y | Y | No | Yes |
| ZNF485 |  | chr10:43421860-43433357 | N | Y | Y | Y | No | Yes |
| RTN2 |  | chr19:50680389-50692151 | N | Y | Y | Y | No | Yes |
| CYP1A1 | NM_014835 | chr15:72798935-72804930 | N | Y | Y | Y | No | Yes |
| FOXO1 |  | chr13:40027800-40138734 | N | Y | Y | Y | No | Yes |
| TNIK |  | chr3:172262985-172660891 | N | Y | Y | Y | No | Yes |
| FZD8 |  | chr10:35967182-35970368 | N | Y | Y | Y | No | Yes |
| HEG1 |  | chr3:126167243-126257492 | N | Y | Y | Y | No | Yes |
| ATP7B | NM_001171138 | chr13:51404805-51483631 | N | Y | Y | Y | No | Yes |
| HIVEP3 |  | chr1:41748270-42157083 | N | Y | Y | Y | No | Yes |
| ANKRD18A | NM_001098621 | chr9:38561362-38610360 | N | Y | Y | Y | No | Yes |
| C11orf45 | NM_001143775 | chr11:128274669-128280802 | N | Y | Y | Y | No | Yes |
| NUAK2 |  | chr1:203537813-203557506 | N | Y | Y | Y | No | Yes |
| BCL11B | NM_001327 | chr14:98705377-98807575 | N | Y | Y | Y | No | Yes |
| RAB19 |  | chr7:139750311-139772519 | N | Y | Y | Y | No | Yes |
| NOG |  | chr17:52026058-52027950 | N | Y | Y | Y | No | Yes |
| GPR160 |  | chr3:171238428-171285875 | N | Y | Y | Y | No | Yes |
| ODZ4 |  | chr11:78041975-78829343 | N | Y | Y | Y | No | Yes |
| THRB |  | chr3:24133648-24511317 | N | Y | Y | Y | No | Yes |
| MATN2 |  | chr8:98950486-99118122 | N | Y | Y | Y | No | Yes |
| CGB7 | NM_016350 | chr19:54249342-54250809 | N | Y | Y | Y | No | Yes |
| PLEKHG1 |  | chr6:150962691-151206492 | N | Y | Y | Y | No | Yes |
| ANKRD56 | NR_028502 | chr4:78035105-78038026 | N | Y | Y | Y | No | Yes |
| ABTB2 | NM_004301 | chr11:34129110-34335378 | N | Y | Y | Y | No | Yes |
| TTC9 |  | chr14:70178256-70211830 | N | Y | Y | Y | No | Yes |
| ZNF502 |  | chr3:44729138-44740325 | N | Y | Y | Y | No | Yes |
| ATP12A | NM_017955 | chr13:24152694-24183918 | N | Y | Y | Y | No | Yes |
| BAG3 | NM_001168647 | chr10:121400871-121427319 | N | Y | Y | Y | No | Yes |
| ENGASE | NM_001142310 | chr17:74582613-74596276 | N | Y | Y | Y | No | Yes |
| C17orf28 | NM_001171132 | chr17:70458433-70480495 | N | Y | Y | Y | No | Yes |
| ZBTB10 |  | chr8:81561002-81597165 | N | Y | Y | Y | No | Yes |
| SLC22A23 |  | chr6:3214206-3401792 | N | Y | Y | Y | No | Yes |
| DNAJB5 | NM_001130082 | chr9:34979724-34988428 | N | Y | Y | Y | No | Yes |
| GSTM4 |  | chr1:110000220-110005853 | N | Y | Y | Y | No | Yes |
| RBP7 |  | chr1:9979841-9998664 | N | Y | Y | Y | No | Yes |
| SPOCK3 |  | chr4:167891110-168392316 | N | Y | Y | Y | No | Yes |
| SMOX |  | chr20:4077449-4116369 | N | Y | Y | Y | No | Yes |
| TBKBP1 |  | chr17:43127628-43144426 | N | Y | Y | Y | No | Yes |
| VLDLR |  | chr9:2611792-2644485 | N | Y | Y | Y | No | Yes |
| SLC27A2 |  | chr15:48261684-48315880 | N | Y | Y | Y | No | Yes |
| PLA2G7 |  | chr6:46780012-46811389 | N | Y | Y | Y | No | Yes |
| GREB1L |  | chr18:17076200-17356787 | N | Y | Y | Y | No | Yes |
| GATA6 |  | chr18:18003413-18036225 | N | Y | Y | Y | No | Yes |
| BTC | NM_001114397 | chr4:75890471-75938906 | N | Y | Y | Y | No | Yes |
| FLJ13224 |  | chr12:31368516-31370146 | N | Y | Y | Y | No | Yes |
| DTX4 | NM_006415 | chr11:58696387-58732636 | N | Y | Y | Y | No | Yes |
| DMRTA1 | NM_001042663 | chr9:22436839-22442472 | N | Y | Y | Y | No | Yes |
| CLDN23 | NM_017670 | chr8:8597075-8599027 | N | Y | Y | Y | No | Yes |
| JMJD5 |  | chr16:27122796-27140590 | N | Y | Y | Y | No | Yes |
| TMEM220 |  | chr17:10557363-10574371 | N | Y | Y | Y | No | Yes |
| CACHD1 | NM_001513 | chr1:64709063-64931329 | N | Y | Y | Y | No | Yes |
| KCNC4 |  | chr1:110555587-110578189 | N | Y | Y | Y | No | Yes |
| C3orf57 | NM_001099414 | chr3:162545273-162572565 | N | Y | Y | Y | No | Yes |
| ZNF503 |  | chr10:76827610-76831519 | N | Y | Y | Y | No | Yes |
| RND1 |  | chr12:47537194-47545920 | N | Y | Y | Y | No | Yes |
| RARRES1 |  | chr3:159905133-159932969 | N | Y | Y | Y | No | Yes |
| CABLES1 | NM_001079684 | chr18:18969724-19094432 | N | Y | Y | Y | No | Yes |
| BOC | NM_022779 | chr3:114414064-114488995 | N | Y | Y | Y | No | Yes |
| ZNF703 |  | chr8:37672458-37675554 | N | Y | Y | Y | No | Yes |
| HEY1 |  | chr8:80838799-80842653 | N | Y | Y | Y | No | Yes |
| LAMP3 |  | chr3:184322696-184363361 | N | Y | Y | Y | No | Yes |
| PPM1E |  | chr17:54188230-54417316 | N | Y | Y | Y | No | Yes |
| KAZALD1 |  | chr10:102810988-102815340 | N | Y | Y | Y | No | Yes |
| DFNB31 | NM_002626 | chr9:116204181-116305316 | N | Y | Y | Y | No | Yes |
| FAM131B | NM_001042494 | chr7:142760614-142769962 | N | Y | Y | Y | No | Yes |
| MDH1B |  | chr2:207310733-207338295 | N | Y | Y | Y | No | Yes |
| MAML3 |  | chr4:140856995-141294683 | N | Y | Y | Y | No | Yes |
| BCL11A | NM_139250 | chr2:60531805-60634137 | N | Y | Y | Y | No | Yes |
| IL20RA |  | chr6:137362800-137407991 | N | Y | Y | Y | No | Yes |
| TTC18 |  | chr10:74683522-74788623 | N | Y | Y | Y | No | Yes |
| CDON | NM_079420 | chr11:125331922-125438397 | N | Y | Y | Y | No | Yes |
| SLC6A20 |  | chr3:45771944-45813039 | N | Y | Y | Y | No | Yes |
| C1QTNF2 | NM_000819 | chr5:159707352-159730226 | N | Y | Y | Y | No | Yes |
| TCF7L1 |  | chr2:85214244-85391016 | N | Y | Y | Y | No | Yes |
| RAET1K |  | chr6:150360847-150367973 | N | Y | Y | Y | No | Yes |
| C20orf132 | NM_005261 | chr20:35163042-35241388 | N | Y | Y | Y | No | Yes |
| FLJ35024 |  | chr9:2525654-2612373 | N | Y | Y | Y | No | Yes |
| C8orf47 | NM_001098721 | chr8:99145925-99175014 | N | Y | Y | Y | No | Yes |
| FLJ13197 |  | chr4:38290716-38342644 | N | Y | Y | Y | No | Yes |
| CCDC110 | NM_001167985 | chr4:186603329-186629907 | N | Y | Y | Y | No | Yes |
| ST8SIA1 |  | chr12:22237591-22378915 | N | Y | Y | Y | No | Yes |
| TLL2 |  | chr10:98114355-98263658 | N | Y | Y | Y | No | Yes |
| MMP25 |  | chr16:3036682-3050725 | N | Y | Y | Y | No | Yes |
| MAPT |  | chr17:41327543-41461546 | N | Y | Y | Y | No | Yes |
| DNAI1 | NM_001128429 | chr9:34448810-34510982 | N | Y | Y | Y | No | Yes |
| TRPC4 |  | chr13:37108774-37341939 | N | Y | Y | Y | No | Yes |
| ADAMTS3 | NM_001130524 | chr4:73365550-73653380 | N | Y | Y | Y | No | Yes |
| SLC16A14 |  | chr2:230607941-230641863 | N | Y | Y | Y | No | Yes |
| EFCAB6 | NM_005681 | chr22:42255986-42539451 | N | Y | Y | Y | No | Yes |
| OTX1 |  | chr2:63131468-63137816 | N | Y | Y | Y | No | Yes |
| MDGA1 |  | chr6:37708261-37773744 | N | Y | Y | Y | No | Yes |
| MAN1C1 |  | chr1:25816545-25983845 | N | Y | Y | Y | No | Yes |
| KIAA1257 |  | chr3:130172473-130195676 | N | Y | Y | Y | No | Yes |
| VWA3B |  | chr2:98070026-98295842 | N | Y | Y | Y | No | Yes |
| POM121L2 |  | chr6:27384820-27387990 | N | Y | Y | Y | No | Yes |
| ELMOD1 | NM_016271 | chr11:106967026-107042715 | N | Y | Y | Y | No | Yes |
| LOC643923 |  | chr11:106967680-106969159 | N | Y | Y | Y | No | Yes |
| RBM20 |  | chr10:112394144-112589217 | N | Y | Y | Y | No | Yes |
| VWA5B2 |  | chr3:185431010-185442810 | N | Y | Y | Y | No | Yes |
| SLCO5A1 |  | chr8:70747123-70908179 | N | Y | Y | Y | No | Yes |
| ST8SIA6 |  | chr10:17402681-17536260 | N | Y | Y | Y | No | Yes |
| SCNN1G |  | chr16:23101540-23135701 | N | Y | Y | Y | No | Yes |
| NEUROG2 |  | chr4:113654120-113656777 | N | Y | Y | Y | No | Yes |
| HFM1 |  | chr1:91498910-91643014 | N | Y | Y | Y | No | Yes |
| LOC100128977 |  | chr17:41276503-41328675 | N | Y | Y | Y | No | Yes |
| LOC100130148 |  | chr17:41328944-41331959 | N | Y | Y | Y | No | Yes |
| ASTN2 | NM_001145306 | chr9:118227327-119217138 | Y | N | Y | Y | No | Yes |
| HOXA11AS |  | chr7:27191551-27195437 | Y | N | Y | Y | No | Yes |
| LOC441046 |  | chr4:144700074-144702062 | Y | N | Y | Y | No | Yes |
| MIR196A1 |  | chr17:44064850-44064920 | Y | N | Y | Y | No | Yes |
| MIR618 |  | chr12:79853646-79853743 | Y | N | Y | Y | No | Yes |
| CDH2 | NM_002441 | chr18:23784927-24011443 | Y | N | Y | Y | No | Yes |
| PUS3 |  | chr11:125268590-125278326 | Y | N | Y | Y | No | Yes |
| CBX4 | NM_001007225 | chr17:75421549-75427808 | Y | N | Y | Y | No | Yes |
| CD40 | NR_031680 | chr20:44180312-44191791 | Y | N | Y | Y | No | Yes |
| IQSEC2 |  | chrX:53278789-53367247 | Y | N | Y | Y | No | Yes |
| H2AFY2 |  | chr10:71482362-71542046 | Y | N | Y | Y | No | Yes |
| ZNF232 |  | chr17:4949754-4967121 | Y | N | Y | Y | No | Yes |
| ST6GALNAC5 |  | chr1:77105773-77302325 | Y | N | Y | Y | No | Yes |
| SHISA2 |  | chr13:25516734-25523198 | Y | N | Y | Y | No | Yes |
| NEFL |  | chr8:24864387-24870048 | Y | N | Y | Y | No | Yes |
| EPB41L4A | NM_001014764 | chr5:111526213-111782909 | Y | N | Y | Y | No | Yes |
| LEF1 |  | chr4:109188149-109309561 | Y | N | Y | Y | No | Yes |
| BMP6 | NM_001165928 | chr6:7672009-7826960 | Y | N | Y | Y | No | Yes |
| SHISA9 |  | chr16:12902977-13241773 | Y | N | Y | Y | No | Yes |
| MSX1 |  | chr4:4912292-4916561 | Y | N | Y | Y | No | Yes |
| ACSS1 | NM_001102406 | chr20:24934872-24987616 | Y | N | Y | Y | No | Yes |
| STOX2 |  | chr4:185063502-185175869 | Y | N | Y | Y | No | Yes |
| CLEC11A | NM_001017973 | chr19:55918416-55920793 | Y | N | Y | Y | No | Yes |
| TMEFF2 |  | chr2:192522991-192767889 | Y | N | Y | Y | No | Yes |
| CCDC85A | NM_001001851 | chr2:56264761-56466813 | Y | N | Y | Y | No | Yes |
| AUTS2 | NM_001037144 | chr7:68701840-69895411 | Y | N | Y | Y | No | Yes |
| ATHL1 | NM_001024736 | chr11:279137-285688 | Y | N | Y | Y | No | Yes |
| ASRGL1 | NM_001039803 | chr11:61861349-61917463 | Y | N | Y | Y | No | Yes |
| FAM105A | NM_001024666 | chr5:14634890-14669285 | Y | N | Y | Y | No | Yes |
| CCDC147 | NM_001127389 | chr10:106103511-106204838 | Y | N | Y | Y | No | Yes |
| CYFIP2 | NM_001011708 | chr5:156625764-156755184 | Y | N | Y | Y | No | Yes |
| IQGAP2 |  | chr5:75734904-76039713 | Y | N | Y | Y | No | Yes |
| C14orf37 | NM_003686 | chr14:57540560-57688600 | Y | N | Y | Y | No | Yes |
| PPAPDC1A |  | chr10:122206455-122339357 | Y | N | Y | Y | No | Yes |
| EPDR1 | NM_001039770 | chr7:37926687-37958067 | Y | N | Y | Y | No | Yes |
| PLEKHN1 |  | chr1:891739-900345 | Y | N | Y | Y | No | Yes |
| ISM1 |  | chr20:13150417-13229297 | Y | N | Y | Y | No | Yes |
| CRYBA2 | NM_001142353 | chr2:219563155-219566365 | Y | N | Y | Y | No | Yes |
| GUCY1A3 |  | chr4:156807311-156872951 | Y | N | Y | Y | No | Yes |
| SLC4A4 |  | chr4:72271866-72656667 | Y | N | Y | Y | No | Yes |
| FOXA1 |  | chr14:37128941-37134240 | Y | N | Y | Y | No | Yes |
| SEMA6A |  | chr5:115807149-115938450 | Y | N | Y | Y | No | Yes |
| HOXA11 |  | chr7:27187300-27191360 | Y | N | Y | Y | No | Yes |
| SAMD5 |  | chr6:147871755-147932850 | Y | N | Y | Y | No | Yes |
| NTRK2 |  | chr9:86474445-86620441 | Y | N | Y | Y | No | Yes |
| IGF2BP1 |  | chr17:44429772-44488504 | Y | N | Y | Y | No | Yes |
| SLC30A3 |  | chr2:27330943-27339464 | Y | N | Y | Y | No | Yes |
| STXBP5L |  | chr3:122109739-122626298 | Y | N | Y | Y | No | Yes |
| LOC645323 |  | chr5:87872352-88016376 | Y | N | Y | Y | No | Yes |
| RIMS4 |  | chr20:42813862-42872326 | Y | N | Y | Y | No | Yes |
| MMP16 |  | chr8:89118575-89408833 | Y | N | Y | Y | No | Yes |
| KIF5C |  | chr2:149349288-149591519 | Y | N | Y | Y | No | Yes |
| KIAA1383 |  | chr1:231007260-231012715 | Y | N | Y | Y | No | Yes |
| SLC27A6 |  | chr5:128329111-128397234 | Y | N | Y | Y | No | Yes |
| TMEM37 |  | chr2:119905915-119912566 | Y | N | Y | Y | No | Yes |
| CACNB4 | NM_014511 | chr2:152397533-152663839 | Y | N | Y | Y | No | Yes |
| SFMBT2 |  | chr10:7244254-7493456 | Y | N | Y | Y | No | Yes |
| ADAM23 | NM_020140 | chr2:207016612-207190924 | Y | N | Y | Y | No | Yes |
| EBF2 | NM_004605 | chr8:25757489-25958309 | Y | N | Y | Y | No | Yes |
| FGF13 |  | chrX:137541401-138114851 | Y | N | Y | Y | No | Yes |
| NCRNA00092 |  | chr9:97821836-97823858 | Y | N | Y | Y | No | Yes |
| STK32B |  | chr4:5104427-5553626 | Y | N | Y | Y | No | Yes |
| ZNF704 |  | chr8:81713323-81949571 | Y | N | Y | Y | No | Yes |
| SLITRK2 |  | chrX:144707038-144715050 | Y | N | Y | Y | No | Yes |
| FAM20A | NM_017767 | chr17:64042852-64108690 | Y | N | Y | Y | No | Yes |
| SNTB1 |  | chr8:121617166-121893490 | Y | N | Y | Y | No | Yes |
| PHOSPHO1 |  | chr17:44655730-44663127 | Y | N | Y | Y | No | Yes |
| MED12L |  | chr3:152287365-152634500 | Y | N | Y | Y | No | Yes |
| PKDCC |  | chr2:42128664-42139170 | Y | N | Y | Y | No | Yes |
| CACNG8 | NM_001130107 | chr19:59158105-59177951 | Y | N | Y | Y | No | Yes |
| LOC401097 |  | chr3:161426116-161428693 | Y | N | Y | Y | No | Yes |
| FOXE1 |  | chr9:99655357-99658818 | Y | N | Y | Y | No | Yes |
| PCDH19 |  | chrX:99433297-99551927 | Y | N | Y | Y | No | Yes |
| HOXB5 |  | chr17:44023617-44026102 | Y | N | Y | Y | No | Yes |
| NKX6-1 |  | chr4:85633459-85638411 | Y | N | Y | Y | No | Yes |
| C6orf176 | NM_001001560 | chr6:166272938-166321517 | Y | N | Y | Y | No | Yes |
| LPPR5 |  | chr1:99128388-99243037 | Y | N | Y | Y | No | Yes |
| NTNG2 |  | chr9:134027154-134108041 | Y | N | Y | Y | No | Yes |
| GFRA1 |  | chr10:117806433-118022786 | Y | N | Y | Y | No | Yes |
| NR3C2 |  | chr4:149219365-149583122 | Y | N | Y | Y | No | Yes |
| HOXA13 |  | chr7:27203023-27206250 | Y | N | Y | Y | No | Yes |
| C12orf68 | NM_001164623 | chr12:46863632-46865976 | Y | N | Y | Y | No | Yes |
| ROR2 |  | chr9:93524704-93752265 | Y | N | Y | Y | No | Yes |
| TRIM71 |  | chr3:32834513-32908775 | Y | N | Y | Y | No | Yes |
| KCNJ6 |  | chr21:37918656-38210566 | Y | N | Y | Y | No | Yes |
| CCK | NM_005365 | chr3:42274321-42282666 | Y | N | Y | Y | No | Yes |
| PRKCB |  | chr16:23754800-24139431 | Y | N | Y | Y | No | Yes |
| EGFLAM | NM_001100588 | chr5:38294289-38500407 | Y | N | Y | Y | No | Yes |
| SLC16A9 |  | chr10:61080528-61139655 | Y | N | Y | Y | No | Yes |
| APBA1 | NM_001039693 | chr9:71232268-71477095 | Y | N | Y | Y | No | Yes |
| TRIL |  | chr7:28959498-28964554 | Y | N | Y | Y | No | Yes |
| LOC285548 |  | chr4:13156797-13158546 | Y | N | Y | Y | No | Yes |
| FOXF1 |  | chr16:85101633-85105571 | Y | N | Y | Y | No | Yes |
| ADAMTS9 | NM_001134296 | chr3:64476370-64648405 | Y | N | Y | Y | No | Yes |
| NPR1 |  | chr1:151917787-151933092 | Y | N | Y | Y | No | Yes |
| KCTD8 |  | chr4:43870678-44145581 | Y | N | Y | Y | No | Yes |
| FOXD3 |  | chr1:63561317-63563385 | Y | N | Y | Y | No | Yes |
| PARM1 |  | chr4:76077321-76194347 | Y | N | Y | Y | No | Yes |
| NPAS3 |  | chr14:32478209-33343132 | Y | N | Y | Y | No | Yes |
| NXPH1 |  | chr7:8440109-8759118 | Y | N | Y | Y | No | Yes |
| KIAA1024 |  | chr15:77511912-77551697 | Y | N | Y | Y | No | Yes |
| DOCK3 | NR_003358 | chr3:50687675-51396669 | Y | N | Y | Y | No | Yes |
| ATOH1 | NM_001024844 | chr4:94969100-94970165 | Y | N | Y | Y | No | Yes |
| GADD45G |  | chr9:91409746-91411287 | Y | N | Y | Y | No | Yes |
| SP5 |  | chr2:171280102-171282743 | Y | N | Y | Y | No | Yes |
| CELF2 | NM_001143944 | chr10:11246998-11418678 | Y | N | Y | Y | No | Yes |
| PDE3A |  | chr12:20413463-20725148 | Y | N | Y | Y | No | Yes |
| SLITRK3 |  | chr3:166387201-166397163 | Y | N | Y | Y | No | Yes |
| SLC6A4 |  | chr17:25547505-25587080 | Y | N | Y | Y | No | Yes |
| CNR1 | NM_000245 | chr6:88906305-88932486 | Y | N | Y | Y | No | Yes |
| LIN7A |  | chr12:79715301-79855825 | Y | N | Y | Y | No | Yes |
| SSTR1 |  | chr14:37746954-37752019 | Y | N | Y | Y | No | Yes |
| LOC440925 |  | chr2:171277194-171279323 | Y | N | Y | Y | No | Yes |
| SGSM1 |  | chr22:23532135-23652813 | Y | N | Y | Y | No | Yes |
| IHH |  | chr2:219627386-219633482 | Y | N | Y | Y | No | Yes |
| HS6ST3 |  | chr13:95541093-96289813 | Y | N | Y | Y | No | Yes |
| SALL1 |  | chr16:49727386-49742009 | Y | N | Y | Y | No | Yes |
| WIPF1 |  | chr2:175132547-175255873 | Y | N | Y | Y | No | Yes |
| KL |  | chr13:32488570-32538281 | Y | N | Y | Y | No | Yes |
| ZIC2 |  | chr13:99432319-99437020 | Y | N | Y | Y | No | Yes |
| ALX4 | NM_001012270 | chr11:44238853-44288292 | Y | N | Y | Y | No | Yes |
| CDO1 | NM_001146225 | chr5:115168328-115180304 | Y | N | Y | Y | No | Yes |
| VAT1L |  | chr16:76379983-76571502 | Y | N | Y | Y | No | Yes |
| LOC440461 |  | chr17:63706395-63708031 | Y | N | Y | Y | No | Yes |
| VSTM2A |  | chr7:54577512-54604442 | Y | N | Y | Y | No | Yes |
| CLGN | NM_004834 | chr4:141529056-141568265 | Y | N | Y | Y | No | Yes |
| SLC26A4 |  | chr7:107088315-107145488 | Y | N | Y | Y | No | Yes |
| EPHB1 | NM_001076674 | chr3:135996788-136461995 | Y | N | Y | Y | No | Yes |
| TBR1 |  | chr2:161980865-161989819 | Y | N | Y | Y | No | Yes |
| KCNH5 |  | chr14:62243697-62581708 | Y | N | Y | Y | No | Yes |
| SOX21 |  | chr13:94159879-94162390 | Y | N | Y | Y | No | Yes |
| KCNK13 |  | chr14:89597860-89721948 | Y | N | Y | Y | No | Yes |
| LINGO1 |  | chr15:75692423-75711764 | Y | N | Y | Y | No | Yes |
| NKAIN3 |  | chr8:63324054-64066182 | Y | N | Y | Y | No | Yes |
| EDIL3 | NM_001163544 | chr5:83273881-83716367 | Y | N | Y | Y | No | Yes |
| ILDR2 |  | chr1:165154619-165211185 | Y | N | Y | Y | No | Yes |
| EPHA7 | NM_183049 | chr6:94006458-94186021 | Y | N | Y | Y | No | Yes |
| GPR83 |  | chr11:93750124-93774233 | Y | N | Y | Y | No | Yes |
| PHF21B |  | chr22:43655708-43784473 | Y | N | Y | Y | No | Yes |
| SYT6 |  | chr1:114433436-114497995 | Y | N | Y | Y | No | Yes |
| FOXB1 |  | chr15:58083712-58085434 | Y | N | Y | Y | No | Yes |
| GFI1 |  | chr1:92712905-92721944 | Y | N | Y | Y | No | Yes |
| PGR |  | chr11:100405564-100505754 | Y | N | Y | Y | No | Yes |
| LONRF2 |  | chr2:100256184-100305627 | Y | N | Y | Y | No | Yes |
| GALNTL6 |  | chr4:172971149-174198133 | Y | N | Y | Y | No | Yes |
| NOVA1 |  | chr14:25984928-26136800 | Y | N | Y | Y | No | Yes |
| VGLL2 |  | chr6:117693413-117701421 | Y | N | Y | Y | No | Yes |
| IGDCC4 |  | chr15:63460877-63502463 | Y | N | Y | Y | No | Yes |
| FAM150A | NM_153453 | chr8:53609150-53640574 | Y | N | Y | Y | No | Yes |
| WSCD1 |  | chr17:5914657-5968471 | Y | N | Y | Y | No | Yes |
| PITX2 |  | chr4:111758028-111763703 | Y | N | Y | Y | No | Yes |
| CACNA1C | NM_001129993 | chr12:2032676-2677376 | Y | N | Y | Y | No | Yes |
| NKX2-6 |  | chr8:23615909-23619867 | Y | N | Y | Y | No | Yes |
| TNFRSF11B |  | chr8:120004976-120033564 | Y | N | Y | Y | No | Yes |
| GLB1L3 |  | chr11:133651484-133694668 | Y | N | Y | Y | No | Yes |
| TMEM26 |  | chr10:62836406-62883214 | Y | N | Y | Y | No | Yes |
| GUCY1A2 |  | chr11:106063119-106394381 | Y | N | Y | Y | No | Yes |
| GPR150 |  | chr5:94981735-94983040 | Y | N | Y | Y | No | Yes |
| FIGNL2 |  | chr12:50497942-50502475 | Y | N | Y | Y | No | Yes |
| KCNJ10 |  | chr1:158274656-158306585 | Y | N | Y | Y | No | Yes |
| LOC286002 |  | chr7:107084196-107089479 | Y | N | Y | Y | No | Yes |
| KIF26B |  | chr1:243384909-243933051 | Y | N | Y | Y | No | Yes |
| FOXA3 |  | chr19:51059357-51068895 | Y | N | Y | Y | No | Yes |
| PCDH17 |  | chr13:57103789-57201066 | Y | N | Y | Y | No | Yes |
| SV2B |  | chr15:89444185-89645542 | Y | N | Y | Y | No | Yes |
| IRF8 |  | chr16:84490274-84513712 | Y | N | Y | Y | No | Yes |
| DPYSL5 | NM_145662 | chr2:26924472-27026723 | Y | N | Y | Y | No | Yes |
| LRRC16B |  | chr14:23591045-23608777 | Y | N | Y | Y | No | Yes |
| ISL1 |  | chr5:50714714-50726320 | Y | N | Y | Y | No | Yes |
| FAM78B | NM_003076 | chr1:164305880-164402582 | Y | N | Y | Y | No | Yes |
| BARHL2 | NM_001127457 | chr1:90950166-90955382 | Y | N | Y | Y | No | Yes |
| ADCY8 | NM_030882 | chr8:131861728-132122017 | Y | N | Y | Y | No | Yes |
| POU3F3 |  | chr2:104838400-104839903 | Y | N | Y | Y | No | Yes |
| PDE3B |  | chr11:14621844-14850178 | Y | N | Y | Y | No | Yes |
| WNT2 |  | chr7:116703923-116750579 | Y | N | Y | Y | No | Yes |
| NLGN1 |  | chr3:174598937-175483810 | Y | N | Y | Y | No | Yes |
| ARMC4 | NM_001156474 | chr10:28141102-28327983 | Y | N | Y | Y | No | Yes |
| ZNF521 |  | chr18:20895885-21186212 | Y | N | Y | Y | No | Yes |
| RSPO1 |  | chr1:37849537-37873078 | Y | N | Y | Y | No | Yes |
| ASXL3 | NM_001134375 | chr18:29412538-29581397 | Y | N | Y | Y | No | Yes |
| CSMD1 | NM_024870 | chr8:2780282-4839736 | Y | N | Y | Y | No | Yes |
| LPPR4 |  | chr1:99502435-99547724 | Y | N | Y | Y | No | Yes |
| ADCY2 | NM_001006634 | chr5:7449342-7883194 | Y | N | Y | Y | No | Yes |
| GRM3 |  | chr7:86111165-86332128 | Y | N | Y | Y | No | Yes |
| GPR88 |  | chr1:100776315-100780171 | Y | N | Y | Y | No | Yes |
| ERBB4 | NM_000546 | chr2:211948686-213111597 | Y | N | Y | Y | No | Yes |
| C4orf31 | NM_001530 | chr4:122176231-122213123 | Y | N | Y | Y | No | Yes |
| RPRML |  | chr17:42410521-42411613 | Y | N | Y | Y | No | Yes |
| LOC441177 |  | chr6:166321028-166323093 | Y | N | Y | Y | No | Yes |
| NKX3-2 |  | chr4:13151551-13155212 | Y | N | Y | Y | No | Yes |
| WNT1 |  | chr12:47658502-47662662 | Y | N | Y | Y | No | Yes |
| CPXM1 | NM_012230 | chr20:2722714-2729282 | Y | N | Y | Y | No | Yes |
| MKX |  | chr10:28001808-28074784 | Y | N | Y | Y | No | Yes |
| TRPC6 |  | chr11:100827504-100959869 | Y | N | Y | Y | No | Yes |
| HOXD9 |  | chr2:176695658-176697891 | Y | N | Y | Y | No | Yes |
| CLVS2 | NM_018899 | chr6:123359280-123426762 | Y | N | Y | Y | No | Yes |
| ARHGAP20 | NM_001105530 | chr11:109952975-110088661 | Y | N | Y | Y | No | Yes |
| ABCC8 | NM_003474 | chr11:17371007-17455025 | Y | N | Y | Y | No | Yes |
| LGR5 |  | chr12:70120079-70264888 | Y | N | Y | Y | No | Yes |
| INSM2 |  | chr14:35072998-35076011 | Y | N | Y | Y | No | Yes |
| CCDC140 | NR_027354 | chr2:222871109-222878180 | Y | N | Y | Y | No | Yes |
| OXGR1 |  | chr13:96435973-96444605 | Y | N | Y | Y | No | Yes |
| FLJ11235 |  | chr5:111783178-111784572 | Y | N | Y | Y | No | Yes |
| OR3A4 |  | chr17:3160288-3161490 | Y | N | Y | Y | No | Yes |
| MIR9-3 |  | chr15:87712251-87712340 | Y | N | Y | Y | No | Yes |
| MESTIT1 |  | chr7:129914133-129918249 | N | N | Y | Y | No | Yes |
| GGTA1 |  | chr9:123257139-123302127 | N | N | Y | Y | No | Yes |
| PTPN3 |  | chr9:111177794-111300414 | N | N | Y | Y | No | Yes |
| MEST |  | chr7:129919173-129933367 | N | N | Y | Y | No | Yes |
| APOBEC3B | NM_001093755 | chr22:37708350-37718729 | N | N | Y | Y | No | Yes |
| MSL3L2 |  | chr2:234438826-234441794 | N | N | Y | Y | No | Yes |
| SFRS13B |  | chr6:89862396-89884519 | N | N | Y | Y | No | Yes |
| MIR148A |  | chr7:25956063-25956131 | N | N | Y | Y | No | Yes |
| CDK4 | NM_001135091 | chr12:56428269-56432431 | N | N | Y | Y | No | Yes |
| PIH1D1 |  | chr19:54641361-54646927 | N | N | Y | Y | No | Yes |
| ILKAP |  | chr2:238743781-238777063 | N | N | Y | Y | No | Yes |
| TMEM181 |  | chr6:158877455-158976455 | N | N | Y | Y | No | Yes |
| KDELR3 |  | chr22:37194028-37209391 | N | N | Y | Y | No | Yes |
| LRRC8D |  | chr1:90059160-90174575 | N | N | Y | Y | No | Yes |
| HCFC1R1 |  | chr16:3012626-3014288 | N | N | Y | Y | No | Yes |
| CD70 | NM_012308 | chr19:6536849-6542163 | N | N | Y | Y | No | Yes |
| LYSMD2 |  | chr15:49802553-49830942 | N | N | Y | Y | No | Yes |
| THOC6 |  | chr16:3014032-3017757 | N | N | Y | Y | No | Yes |
| BAIAP2L1 | NM_001851 | chr7:97758903-97868363 | N | N | Y | Y | No | Yes |
| MAPRE2 |  | chr18:30810889-30976375 | N | N | Y | Y | No | Yes |
| TARBP1 |  | chr1:232593681-232681472 | N | N | Y | Y | No | Yes |
| TTF1 |  | chr9:134240757-134272042 | N | N | Y | Y | No | Yes |
| TMEM128 |  | chr4:4288169-4300835 | N | N | Y | Y | No | Yes |
| KCTD18 |  | chr2:201061928-201083037 | N | N | Y | Y | No | Yes |
| KIAA1147 |  | chr7:141002996-141048422 | N | N | Y | Y | No | Yes |
| KCNMB4 |  | chr12:69046328-69111245 | N | N | Y | Y | No | Yes |
| HPSE |  | chr4:84435491-84475058 | N | N | Y | Y | No | Yes |
| ZDBF2 |  | chr2:206847767-206887393 | N | N | Y | Y | No | Yes |
| IRF2BP1 |  | chr19:51078705-51081216 | N | N | Y | Y | No | Yes |
| TESK1 |  | chr9:35595280-35600038 | N | N | Y | Y | No | Yes |
| MANEAL |  | chr1:38032360-38039865 | N | N | Y | Y | No | Yes |
| TRIM36 |  | chr5:114534698-114544142 | N | N | Y | Y | No | Yes |
| CBX2 | NR_027132 | chr17:75366571-75376044 | N | N | Y | Y | No | Yes |
| ZNF678 |  | chr1:225817842-225916786 | N | N | Y | Y | No | Yes |
| NGFR |  | chr17:44927653-44947371 | N | N | Y | Y | No | Yes |
| NUAK1 |  | chr12:104981254-105057941 | N | N | Y | Y | No | Yes |
| C17orf96 | NM_001001715 | chr17:34081484-34084713 | N | N | Y | Y | No | Yes |
| GPX7 |  | chr1:52840630-52847310 | N | N | Y | Y | No | Yes |
| MLYCD |  | chr16:82490230-82507288 | N | N | Y | Y | No | Yes |
| C8orf39 | NM_020748 | chr8:94821514-94822223 | N | N | Y | Y | No | Yes |
| SLC1A4 |  | chr2:65069082-65104501 | N | N | Y | Y | No | Yes |
| CPM | NM_032882 | chr12:67531224-67613246 | N | N | Y | Y | No | Yes |
| DHRS12 | NM_001135862 | chr13:51242920-51276235 | N | N | Y | Y | No | Yes |
| EML5 | NM_001017395 | chr14:88150926-88328849 | N | N | Y | Y | No | Yes |
| GATM |  | chr15:43440613-43458272 | N | N | Y | Y | No | Yes |
| DHRS4L1 | NM_005983 | chr14:23575549-23590420 | N | N | Y | Y | No | Yes |
| BMP4 | NM_001079862 | chr14:53486204-53493304 | N | N | Y | Y | No | Yes |
| C6orf168 | NM_001145143 | chr6:99827513-99904252 | N | N | Y | Y | No | Yes |
| RNF175 |  | chr4:154850761-154900837 | N | N | Y | Y | No | Yes |
| ZNF167 |  | chr3:44571716-44588654 | N | N | Y | Y | No | Yes |
| PRKCQ |  | chr10:6509110-6662244 | N | N | Y | Y | No | Yes |
| LHX6 |  | chr9:124004678-124030840 | N | N | Y | Y | No | Yes |
| TMOD2 |  | chr15:49831049-49895848 | N | N | Y | Y | No | Yes |
| C6orf126 | NM_001143828 | chr6:35852369-35855307 | N | N | Y | Y | No | Yes |
| ABAT | NM_001127219 | chr16:8675944-8785933 | N | N | Y | Y | No | Yes |
| YPEL4 |  | chr11:57169135-57173993 | N | N | Y | Y | No | Yes |
| DDO | NM_001161576 | chr6:110820075-110843446 | N | N | Y | Y | No | Yes |
| MEX3B |  | chr15:80121182-80125416 | N | N | Y | Y | No | Yes |
| FAM26F | NM_001105539 | chr6:116889248-116891627 | N | N | Y | Y | No | Yes |
| ADRA1B | NM_001080156 | chr5:159276317-159332595 | N | N | Y | Y | No | Yes |
| SERPING1 |  | chr11:57121602-57138902 | N | N | Y | Y | No | Yes |
| ZNF579 |  | chr19:60780702-60784023 | N | N | Y | Y | No | Yes |
| ZNF763 |  | chr19:11936868-11952198 | N | N | Y | Y | No | Yes |
| RPP25 |  | chr15:73034495-73036828 | N | N | Y | Y | No | Yes |
| MYEF2 |  | chr15:46218920-46257850 | N | N | Y | Y | No | Yes |
| BACH2 | NM_001122633 | chr6:90692968-91063348 | N | N | Y | Y | No | Yes |
| TNFRSF11A |  | chr18:58143527-58204484 | N | N | Y | Y | No | Yes |
| N4BP3 |  | chr5:177473161-177485713 | N | N | Y | Y | No | Yes |
| C7orf46 | NM_021798 | chr7:23686273-23708794 | N | N | Y | Y | No | Yes |
| TMEM200B |  | chr1:29318524-29323008 | N | N | Y | Y | No | Yes |
| RTN4RL1 |  | chr17:1784720-1874928 | N | N | Y | Y | No | Yes |
| ZNF572 |  | chr8:126054719-126060809 | N | N | Y | Y | No | Yes |
| SCN4B |  | chr11:117509303-117528840 | N | N | Y | Y | No | Yes |
| ZNF578 |  | chr19:57648640-57711943 | N | N | Y | Y | No | Yes |
| COCH | NM_001098270 | chr14:30413491-30429573 | N | N | Y | Y | No | Yes |
| VPS37D |  | chr7:72720109-72724376 | N | N | Y | Y | No | Yes |
| FOXD2 |  | chr1:47674275-47678950 | N | N | Y | Y | No | Yes |
| KCNJ5 |  | chr11:128266522-128293161 | N | N | Y | Y | No | Yes |
| DDN | NM_000280 | chr12:47675199-47679355 | N | N | Y | Y | No | Yes |
| MYADM |  | chr19:59064592-59071501 | N | N | Y | Y | No | Yes |
| TMEM86A |  | chr11:18676926-18682908 | N | N | Y | Y | No | Yes |
| CYP26B1 | NM_001144063 | chr2:72209874-72228471 | N | N | Y | Y | No | Yes |
| RASGEF1B |  | chr4:82567242-82612085 | N | N | Y | Y | No | Yes |
| MYB |  | chr6:135544145-135582004 | N | N | Y | Y | No | Yes |
| EGR2 | NM_004414 | chr10:64241765-64246132 | N | N | Y | Y | No | Yes |
| KIAA1199 |  | chr15:78858766-79031054 | N | N | Y | Y | No | Yes |
| TRIM9 |  | chr14:50511730-50632172 | N | N | Y | Y | No | Yes |
| FOXQ1 |  | chr6:1257674-1259993 | N | N | Y | Y | No | Yes |
| LOC387646 |  | chr10:27574787-27581241 | N | N | Y | Y | No | Yes |
| ALDH1L1 | NM_001009811 | chr3:127305097-127382175 | N | N | Y | Y | No | Yes |
| ZNF665 |  | chr19:58358363-58388431 | N | N | Y | Y | No | Yes |
| STK32A |  | chr5:146594771-146743961 | N | N | Y | Y | No | Yes |
| PITX3 |  | chr10:103979935-103991221 | N | N | Y | Y | No | Yes |
| PLCL2 |  | chr3:16901455-17107101 | N | N | Y | Y | No | Yes |
| COLEC12 | NM_017572 | chr18:309355-490729 | N | N | Y | Y | No | Yes |
| EPO | NM_001130413 | chr7:100156358-100159259 | N | N | Y | Y | No | Yes |
| FAM110B | NM_005069 | chr8:59069666-59224831 | N | N | Y | Y | No | Yes |
| SDK1 |  | chr7:3307605-4275157 | N | N | Y | Y | No | Yes |
| CYS1 | NM_001128635 | chr2:10114376-10137989 | N | N | Y | Y | No | Yes |
| C2CD4A | NM_001032394 | chr15:60146467-60150408 | N | N | Y | Y | No | Yes |
| EBF3 | NM_001142272 | chr10:131523536-131652081 | N | N | Y | Y | No | Yes |
| PRRT4 |  | chr7:127777615-127788975 | N | N | Y | Y | No | Yes |
| ZBTB8B |  | chr1:32703244-32726044 | N | N | Y | Y | No | Yes |
| USP44 |  | chr12:94435017-94466751 | N | N | Y | Y | No | Yes |
| CADM2 | NM_001168299 | chr3:85090822-86206267 | N | N | Y | Y | No | Yes |
| REM1 |  | chr20:29526765-29536369 | N | N | Y | Y | No | Yes |
| SLC1A2 |  | chr11:35229327-35397681 | N | N | Y | Y | No | Yes |
| FAM124A | NM_007122 | chr13:50694507-50753617 | N | N | Y | Y | No | Yes |
| ST6GAL2 |  | chr2:106784489-106869042 | N | N | Y | Y | No | Yes |
| RAD21L1 |  | chr20:1154763-1183145 | N | N | Y | Y | No | Yes |
| C1orf229 | NM_001171937 | chr1:245340084-245342342 | N | N | Y | Y | No | Yes |
| CD38 | NR_030180 | chr4:15389028-15459804 | N | N | Y | Y | No | Yes |
| C15orf27 | NM_001166005 | chr15:74139353-74284359 | N | N | Y | Y | No | Yes |
| KCNH8 |  | chr3:19165020-19552139 | N | N | Y | Y | No | Yes |
| LOC145663 |  | chr15:43458189-43459613 | N | N | Y | Y | No | Yes |
| KCNG3 |  | chr2:42522660-42574741 | N | N | Y | Y | No | Yes |
| ESX1 | NM_001099679 | chrX:103381375-103386255 | N | N | Y | Y | No | Yes |
| NELL2 |  | chr12:43188324-43556900 | N | N | Y | Y | No | Yes |
| C9orf171 | NM_002230 | chr9:134275431-134438496 | N | N | Y | Y | No | Yes |
| NKX2-8 |  | chr14:36118966-36121537 | N | N | Y | Y | No | Yes |
| COL9A2 | NM_001042533 | chr1:40538749-40555526 | N | N | Y | Y | No | Yes |
| MOGAT1 |  | chr2:223244700-223282893 | N | N | Y | Y | No | Yes |
| SGCZ |  | chr8:13991743-15140163 | N | N | Y | Y | No | Yes |
| CHRNB4 | NM_001048209 | chr15:76703690-76720642 | N | N | Y | Y | No | Yes |
| MIR153-1 |  | chr2:219867076-219867166 | N | N | Y | Y | No | Yes |
| MIR1258 |  | chr2:180433808-180433880 | N | N | Y | Y | No | Yes |
| TXNRD3 |  | chr3:127808584-127856657 | Y | Y | Y | Y | Yes | Yes |
| CERKL | NM_015509 | chr2:182109647-182230079 | Y | Y | Y | Y | Yes | Yes |
| AP1S1 | NR_024622 | chr7:100584405-100591277 | Y | Y | Y | Y | Yes | Yes |
| SFXN1 |  | chr5:174838119-174888227 | Y | Y | Y | Y | Yes | Yes |
| CCND2 | NM_005368 | chr12:4253162-4284782 | Y | Y | Y | Y | Yes | Yes |
| PFKP |  | chr10:3099751-3168996 | Y | Y | Y | Y | Yes | Yes |
| NOC2L |  | chr1:869445-884542 | Y | Y | Y | Y | Yes | Yes |
| SEC11C |  | chr18:54958104-54977043 | Y | Y | Y | Y | Yes | Yes |
| PYCARD |  | chr16:31120307-31121752 | Y | Y | Y | Y | Yes | Yes |
| PDRG1 |  | chr20:29996418-30003544 | Y | Y | Y | Y | Yes | Yes |
| MED30 |  | chr8:118602145-118621680 | Y | Y | Y | Y | Yes | Yes |
| SYMPK |  | chr19:51010539-51058388 | Y | Y | Y | Y | Yes | Yes |
| TBC1D1 |  | chr4:37569114-37817189 | Y | Y | Y | Y | Yes | Yes |
| CYP1B1 | NM_001128617 | chr2:38148249-38156827 | Y | Y | Y | Y | Yes | Yes |
| LBH |  | chr2:30307900-30336403 | Y | Y | Y | Y | Yes | Yes |
| NRCAM |  | chr7:107575317-107884062 | Y | Y | Y | Y | Yes | Yes |
| PITX1 |  | chr5:134391322-134397863 | Y | Y | Y | Y | Yes | Yes |
| NFKBIE |  | chr6:44333880-44341503 | Y | Y | Y | Y | Yes | Yes |
| DNER | NM_003711 | chr2:229930588-230287530 | Y | Y | Y | Y | Yes | Yes |
| BLMH | NM_001017916 | chr17:25599348-25643200 | Y | Y | Y | Y | Yes | Yes |
| IRX1 |  | chr5:3649167-3654517 | Y | Y | Y | Y | Yes | Yes |
| CDH11 | NM_001012417 | chr16:63538183-63713420 | Y | Y | Y | Y | Yes | Yes |
| FARP1 | NM_001129898 | chr13:97593434-97695559 | Y | Y | Y | Y | Yes | Yes |
| FLI1 |  | chr11:128067598-128188371 | Y | Y | Y | Y | Yes | Yes |
| MSC |  | chr8:72916330-72919285 | Y | Y | Y | Y | Yes | Yes |
| DLEU2 | NM_001143824 | chr13:49454688-49597678 | Y | Y | Y | Y | Yes | Yes |
| MAST4 |  | chr5:65927931-66501178 | Y | Y | Y | Y | Yes | Yes |
| HOXB7 |  | chr17:44039593-44043382 | Y | Y | Y | Y | Yes | Yes |
| KLHL17 |  | chr1:885829-890958 | Y | Y | Y | Y | Yes | Yes |
| UNC5C |  | chr4:96308711-96689185 | Y | Y | Y | Y | Yes | Yes |
| SAMD12 |  | chr8:119270875-119703365 | Y | Y | Y | Y | Yes | Yes |
| ZFP82 |  | chr19:41574700-41601390 | Y | Y | Y | Y | Yes | Yes |
| PNMA2 |  | chr8:26418112-26427400 | Y | Y | Y | Y | Yes | Yes |
| LHX4 |  | chr1:178466064-178510811 | Y | Y | Y | Y | Yes | Yes |
| APCDD1 | NM_001171740 | chr18:10444624-10478698 | Y | Y | Y | Y | Yes | Yes |
| NKX3-1 |  | chr8:23592151-23596395 | Y | Y | Y | Y | Yes | Yes |
| CA8 | NM_001142603 | chr8:61263976-61356508 | Y | Y | Y | Y | Yes | Yes |
| BATF3 | NM_001317 | chr1:210926381-210939950 | Y | Y | Y | Y | Yes | Yes |
| MAFB |  | chr20:38747930-38751290 | Y | Y | Y | Y | Yes | Yes |
| CITED1 | NM_130760 | chrX:71438214-71442489 | Y | Y | Y | Y | Yes | Yes |
| NOV |  | chr8:120497732-120505859 | Y | Y | Y | Y | Yes | Yes |
| LRRC6 |  | chr8:133653628-133756995 | Y | Y | Y | Y | Yes | Yes |
| HOXB9 |  | chr17:44053517-44058834 | Y | Y | Y | Y | Yes | Yes |
| BAMBI | NM_024027 | chr10:29006429-29011874 | Y | Y | Y | Y | Yes | Yes |
| SLC22A17 |  | chr14:22885378-22891920 | Y | Y | Y | Y | Yes | Yes |
| FAM47E | NM_001029939 | chr4:77391876-77423947 | Y | Y | Y | Y | Yes | Yes |
| TMEM170B |  | chr6:11646496-11691743 | Y | Y | Y | Y | Yes | Yes |
| CACNG4 | NM_032123 | chr17:62391474-62459980 | Y | Y | Y | Y | Yes | Yes |
| EFHD1 | NM_182663 | chr2:233206450-233255734 | Y | Y | Y | Y | Yes | Yes |
| LRGUK |  | chr7:133462644-133599473 | Y | Y | Y | Y | Yes | Yes |
| POU3F1 |  | chr1:38282109-38285037 | Y | Y | Y | Y | Yes | Yes |
| VASH2 |  | chr1:211190509-211231548 | Y | Y | Y | Y | Yes | Yes |
| FAM181B | NM_001145265 | chr11:82120693-82122554 | Y | Y | Y | Y | Yes | Yes |
| CNNM1 | NM_002570 | chr10:101078845-101144077 | Y | Y | Y | Y | Yes | Yes |
| LHX9 |  | chr1:196148257-196165896 | Y | Y | Y | Y | Yes | Yes |
| RUNDC3B |  | chr7:87095664-87299548 | Y | Y | Y | Y | Yes | Yes |
| POU3F2 |  | chr6:99389300-99393387 | Y | Y | Y | Y | Yes | Yes |
| PPFIBP2 |  | chr11:7491576-7631567 | Y | Y | Y | Y | Yes | Yes |
| MAP6 |  | chr11:74975610-75057127 | Y | Y | Y | Y | Yes | Yes |
| PPM1H |  | chr12:61324030-61614932 | Y | Y | Y | Y | Yes | Yes |
| MCF2L2 |  | chr3:184378524-184628549 | Y | Y | Y | Y | Yes | Yes |
| CBX8 | NR_015390 | chr17:75382770-75385485 | Y | Y | Y | Y | Yes | Yes |
| CGNL1 | NM_015384 | chr15:55455996-55630213 | Y | Y | Y | Y | Yes | Yes |
| SHOX2 |  | chr3:159296494-159306646 | Y | Y | Y | Y | Yes | Yes |
| KLHL32 |  | chr6:97479216-97695351 | Y | Y | Y | Y | Yes | Yes |
| NCAM1 |  | chr11:112337204-112654368 | Y | Y | Y | Y | Yes | Yes |
| MEGF11 |  | chr15:63974687-64333129 | Y | Y | Y | Y | Yes | Yes |
| SLC35D3 |  | chr6:137285094-137288469 | Y | Y | Y | Y | Yes | Yes |
| PCSK5 |  | chr9:77695379-77998158 | Y | Y | Y | Y | Yes | Yes |
| SMAD9 |  | chr13:36316968-36392409 | Y | Y | Y | Y | Yes | Yes |
| KIT |  | chr4:55218851-55301638 | Y | Y | Y | Y | Yes | Yes |
| CSPG5 | NM_006162 | chr3:47578731-47595363 | Y | Y | Y | Y | Yes | Yes |
| EFR3B | NM_001164803 | chr2:25118476-25235508 | Y | Y | Y | Y | Yes | Yes |
| CHSY3 | NM_012346 | chr5:129268421-129550226 | Y | Y | Y | Y | Yes | Yes |
| ADRB1 | NM_001011722 | chr10:115793795-115796657 | Y | Y | Y | Y | Yes | Yes |
| ITIH5 |  | chr10:7653373-7748940 | Y | Y | Y | Y | Yes | Yes |
| FGF9 |  | chr13:21143214-21176640 | Y | Y | Y | Y | Yes | Yes |
| HEY2 |  | chr6:126112424-126124108 | Y | Y | Y | Y | Yes | Yes |
| NOL4 |  | chr18:29685061-30057444 | Y | Y | Y | Y | Yes | Yes |
| DACH1 | NM_001165032 | chr13:70910098-71339331 | Y | Y | Y | Y | Yes | Yes |
| SIDT1 |  | chr3:114733907-114831112 | Y | Y | Y | Y | Yes | Yes |
| SCN3B |  | chr11:123005104-123030525 | Y | Y | Y | Y | Yes | Yes |
| GLT25D2 |  | chr1:182171588-182273486 | Y | Y | Y | Y | Yes | Yes |
| SOBP |  | chr6:107918009-108089206 | Y | Y | Y | Y | Yes | Yes |
| CELF6 | NM_000433 | chr15:70364121-70399341 | Y | Y | Y | Y | Yes | Yes |
| SEMA5B |  | chr3:124110732-124229266 | Y | Y | Y | Y | Yes | Yes |
| ZSWIM5 |  | chr1:45254662-45444837 | Y | Y | Y | Y | Yes | Yes |
| ATRNL1 | NM_000087 | chr10:116843113-117698486 | Y | Y | Y | Y | Yes | Yes |
| IGSF11 |  | chr3:120102168-120236366 | Y | Y | Y | Y | Yes | Yes |
| PLCL1 |  | chr2:198377670-198722851 | Y | Y | Y | Y | Yes | Yes |
| TRPC3 |  | chr4:123019632-123092359 | Y | Y | Y | Y | Yes | Yes |
| CACNA2D1 | NM_004856 | chr7:81417353-81910967 | Y | Y | Y | Y | Yes | Yes |
| KCND3 |  | chr1:112119976-112333300 | Y | Y | Y | Y | Yes | Yes |
| PROX1 |  | chr1:212228482-212276385 | Y | Y | Y | Y | Yes | Yes |
| IRS4 |  | chrX:107862382-107866263 | Y | Y | Y | Y | Yes | Yes |

Genome-wide ChIP-Seq analyses were previously performed to profile the patterns of H3K4me3 and H3K27me3 in HMLE-vector and HMLE-Twist cells [1]. In order to understand the mechanisms leading to the establishment of bivalent promoters following EMT, the ChIP-Seq data were analyzed to identify bivalent genes. Based on their pre-existing histone modifications in HMLE-vector cells, all bivalent genes in HMLE-Twist cells were classified into four groups: Group I (green highlight), pre-marked with H3K4me3 in HMLE-vector cells that gain H3K27me3; Group II (yellow highlight), pre-marked with H3K27me3 in HMLE-vector cells that gain H3K4me3; Group III (pink highlight), unmarked in HMLE-vector cells that acquire both H3K4me3 and H3K27me3; Group IV (grey highlight), bivalent in HMLE-vector cells with no further change in status. N=No, Y=Yes.

**Supplementary Table 2: Primer sequences used in quantitative RT-PCR or ChIP**

| **Name** | **Sequence** | **Application** |
| --- | --- | --- |
| hBTC RT F | CCTGGGTCTAGTGATCCTTCA | RT-PCR |
| hBTC RT R | CTTTCCGCTTTGATTGTGTGG | RT-PCR |
| hC6orf141 RT F | CCAGAGAGGTGGTTAGGGACT | RT-PCR |
| hC6orf141 RT R | CTCGTTGAAAGACAGAAGGGTAG | RT-PCR |
| hCBX4 F | GCAGAGTGGAGTATCTGGTGA | RT-PCR |
| hCBX4 R | AGCTTGGCACGGTTGTCAG | RT-PCR |
| hCCNA1 RT F | GAGGTCCCGATGCTTGTCAG | RT-PCR |
| hCCNA1 RT R | GTTAGCAGCCCTAGCACTGTC | RT-PCR |
| hCD70 F | GCTTTGGTCCCATTGGTCG | RT-PCR |
| hCD70 R | CGTCCCACCCAAGTGACTC | RT-PCR |
| hCD83 RT F | AAGGGGCAAAATGGTTCTTTCG | RT-PCR |
| hCD83 RT R | GCACCTGTATGTCCCCGAG | RT-PCR |
| hCDH1 RT F | CGAGAGCTACACGTTCACGG | RT-PCR |
| hCDH1 RT R | GGGTGTCGAGGGAAAAATAGG | RT-PCR |
| hCDK4 F | ATGGCTACCTCTCGATATGAGC | RT-PCR |
| hCDK4 R | CATTGGGGACTCTCACACTCT | RT-PCR |
| hCDS1 RT F | AGTTCCTCATTCGCTACCATAGA | RT-PCR |
| hCDS1 RT R | GGTGTGACTGAGTGACAGTTATC | RT-PCR |
| hCGB7 RT F | GGTGTGCAACTACCGCGAT | RT-PCR |
| hCGB7 RT R | GGAGTCGGGATGGACTTGGA | RT-PCR |
| hCOBLL1 RT F | AGACCATAGTGAGAGTGAGTCC | RT-PCR |
| hCOBLL1 RT R | TCTCTGTTGACATCCATCGCATA | RT-PCR |
| hCREG1 RT F | GGCGTGCCCTATTTCTACCTG | RT-PCR |
| hCREG1 RT R | CAAAGTCATGGTCAGTGTAGCAT | RT-PCR |
| hDSC3 RT F | GACCCTCGTGATCTTCAGTCG | RT-PCR |
| hDSC3 RT R | TCACTTGACCGGATGAGGTCT | RT-PCR |
| hFAM117A RT F | GTGGCCCCAGAAAAGTCAGT | RT-PCR |
| hFAM117A RT R | TGGGTGGCCTTGTCATTGG | RT-PCR |
| hFAM60A RT F | CTCCAGTTCTCGATTCACTGAC | RT-PCR |
| hFAM60A RT R | CGAGTCTCATGCAATCCAAAACA | RT-PCR |
| hFKBP4 RT F | GAAGGCGTGCTGAAGGTCAT | RT-PCR |
| hFKBP4 RT R | TGCCATCTAATAGCCAGCCAG | RT-PCR |
| hFOXO1 RT F | TCGTCATAATCTGTCCCTACACA | RT-PCR |
| hFOXO1 RT R | CGGCTTCGGCTCTTAGCAAA | RT-PCR |
| hFRMD4B RT F | ATGGCTTCGGTGTTCATGTGT | RT-PCR |
| hFRMD4B RT R | GCTCCAGTCTCCTATCATCCAG | RT-PCR |
| hFZD8 RT F | ATCGGCTACAACTACACCTACA | RT-PCR |
| hFZD8 RT R | GTACATGCTGCACAGGAAGAA | RT-PCR |
| hGAPDH RT F | GGAGCGAGATCCCTCCAAAAT | RT-PCR |
| hGAPDH RT R | GGCTGTTGTCATACTTCTCATGG | RT-PCR |
| hH2AFY2 F | GCAGGTGTCATCTTTCCAGTG | RT-PCR |
| hH2AFY2 R | CACGCTGATCCGGTACTTGA | RT-PCR |
| hILKAP F | CTAGCAGTGGCGATTCAGGTT | RT-PCR |
| hILKAP R | TCACCGAAGAGGCTTTACAAAC | RT-PCR |
| hIQSEC2 F | CGTGAGGAAGGCACTCGAAG | RT-PCR |
| hIQSEC2 R | AGCGGTCACTCAGGTCTACAG | RT-PCR |
| hKDELR3 F | TCCCAGTCATTGGCCTTTCC | RT-PCR |
| hKDELR3 R | CCAGTTAGCCAGGTAGAGTGC | RT-PCR |
| hLIPG RT F | GGGAGCCCCGTACCTTTTG | RT-PCR |
| hLIPG RT R | CCTCACAGATGGTTTGACCTCA | RT-PCR |
| hLRRC8D F | ATGACATTCAGCCAACTTACCG | RT-PCR |
| hLRRC8D R | TACTGGCAAACAGACCACCTG | RT-PCR |
| hMAP3K9 RT F | GCCGTGTTCGAGTACGAGG | RT-PCR |
| hMAP3K9 RT R | GGACACCTGCGAGTCCTTG | RT-PCR |
| hMAP7 RT F | TCATCATGCCCTACAAAGCTG | RT-PCR |
| hMAP7 RT R | TGCCAGATGTGAGGAAGAGTA | RT-PCR |
| hMARK1 RT F | GAGCGGGACACGGAAAATCAT | RT-PCR |
| hMARK1 RT R | TGCTACTCGACTTGGTAGGCT | RT-PCR |
| hMED30 F | AGCTACAGGATAATCTTCGCCA | RT-PCR |
| hMED30 R | TGGAATAAGTTGCTCGACTGGA | RT-PCR |
| hNEFL F | ATGAGTTCCTTCAGCTACGAGC | RT-PCR |
| hNEFL R | CTGGGCATCAACGATCCAGA | RT-PCR |
| hPDRG1 F | AGGAATCAGAATCGAGAGGGC | RT-PCR |
| hPDRG1 R | CATGTTCCCGAAGCAAACCAT | RT-PCR |
| hPFKP F | GCATGGGTATCTACGTGGGG | RT-PCR |
| hPFKP R | CTCTGCGATGTTTGAGCCTC | RT-PCR |
| hPIH1D1 F | GGGAATGGGGCTAAGCGAG | RT-PCR |
| hPIH1D1 R | TGTCGATTCTGGTCTGGTTGT | RT-PCR |
| hPKP2 RT F | GTGGGCAACGGAAATCTTCAC | RT-PCR |
| hPKP2 RT R | CCAGCCTTTAGCATGTCATAGG | RT-PCR |
| hPUS3 F | AGCGTGCATTTGATTTCAGTGC | RT-PCR |
| hPUS3 R | TGGTATCCCCAGCCCATATAG | RT-PCR |
| hPYCARD F | TGGATGCTCTGTACGGGAAG | RT-PCR |
| hPYCARD R | CCAGGCTGGTGTGAAACTGAA | RT-PCR |
| hRAVER2 RT F | CTGAGCAACCGCAGGAAAATC | RT-PCR |
| hRAVER2 RT R | ACATCTGAATTGCGTTCTGGG | RT-PCR |
| hSEC11C F | CAAGCGCCAGCTCTATTACCA | RT-PCR |
| hSEC11C R | TGTGAGCACGATCAAGCCTTT | RT-PCR |
| hSFXN1 F | TTAACATCAAGGAACCTCGATGG | RT-PCR |
| hSFXN1 R | TGGGGTCAGTTACAGTGAAGA | RT-PCR |
| hSHISA2 F | GGAGACCATCCCCATGATCC | RT-PCR |
| hSHISA2 R | AGCACAGAGAAATTCGTGGGC | RT-PCR |
| hSYMPK F | GAGATCATCGCATTCCAAGCA | RT-PCR |
| hSYMPK R | TCACATTCTCGTCCCTCAAGAG | RT-PCR |
| hTC2N RT F | TGGCTGTACTGAGGATTATTTGC | RT-PCR |
| hTC2N RT R | TGTGAAGGAGTTTCTTGTGTCC | RT-PCR |
| hTGFBI RT F | CACTCTCAAACCTTTACGAGACC | RT-PCR |
| hTGFBI RT R | CGTTGCTAGGGGCGAAGATG | RT-PCR |
| hTHOC6 F | TCCCAGAGCGTCTCACCAT | RT-PCR |
| hTHOC6 R | CCACCGGCTTCTTACTTTCCT | RT-PCR |
| hTMEM181 F | CCTGACCATCTTCGTTGGGAT | RT-PCR |
| hTMEM181 R | CATCGACTTTAACAGTCATGGGA | RT-PCR |
| hZNF232 F | GTGCAGACTAGGATGGCTGTA | RT-PCR |
| hZNF232 R | GCCTGGTCTCATACTCACAAGA | RT-PCR |
| hECadTSS-F | AGCACCATCTCTGGGATGAC | ChIP |
| hECadTSS-R | ATGATTGTGCCACTGCACTC | ChIP |

**References**

1. Malouf GG, Taube JH, Lu Y, Roysarkar T, Panjarian S, Estecio MR, Jelinek J, Yamazaki J, Raynal NJ, Long H, Tahara T, Tinnirello A, Ramachandran P, et al. Architecture of epigenetic reprogramming following Twist1-mediated epithelial-mesenchymal transition. Genome Biol. 2013; 14:R144.
